# Metabolomic Signatures Associated with Radiation-Induced Lung Injury by Correlating Lung Tissue to Plasma in a Rat Model

**DOI:** 10.3390/metabo13091020

**Published:** 2023-09-17

**Authors:** Liming Gu, Wenli Wang, Yifeng Gu, Jianping Cao, Chang Wang

**Affiliations:** State Key Laboratory of Radiation Medicine and Protection, School of Radiation Medicine and Protection, Medical College of Soochow University, School for Radiological and Interdisciplinary Sciences (RAD-X), Jiangsu Provincial Key Laboratory of Radiation Medicine and Protection, Suzhou Industrial Park Ren’ai Road 199, Suzhou 215123, China; 20214020013@stu.suda.edu.cn (L.G.); 20214220035@stu.suda.edu.cn (W.W.); guyifeng19950830@126.com (Y.G.)

**Keywords:** metabolomics, whole-thorax irradiation (WTI), radiation-induced lung injury (RILI), metabolic marker, dynamic

## Abstract

The lung has raised significant concerns because of its radiosensitivity. Radiation-induced lung injury (RILI) has a serious impact on the quality of patients’ lives and limits the effect of radiotherapy on chest tumors. In clinical practice, effective drug intervention for RILI remains to be fully elucidated. Therefore, an in-depth understanding of the biological characteristics is essential to reveal the mechanisms underlying the complex biological processes and discover novel therapeutic targets in RILI. In this study, Wistar rats received 0, 10, 20 or 35 Gy whole-thorax irradiation (WTI). Lung and plasma samples were collected within 5 days post-irradiation. Then, these samples were processed using liquid chromatography–mass spectrometry (LC-MS). A panel of potential plasma metabolic markers was selected by correlation analysis between the lung tissue and plasma metabolic features, followed by the evaluation of radiation injury levels within 5 days following whole-thorax irradiation (WTI). In addition, the multiple metabolic dysregulations primarily involved amino acids, bile acids and lipid and fatty acid β-oxidation-related metabolites, implying disturbances in the urea cycle, intestinal flora metabolism and mitochondrial dysfunction. In particular, the accumulation of long-chain acylcarnitines (ACs) was observed as early as 2 d post-WTI by dynamic plasma metabolic data analysis. Our findings indicate that plasma metabolic markers have the potential for RILI assessment. These results reveal metabolic characteristics following WTI and provide new insights into therapeutic interventions for RILI.

## 1. Introduction

With the development of nuclear power and the widespread application of nuclear technology, the potential risk for radiation damage to people has greatly increased. A rapid, sensitive and accurate assay to assess the severity of the critical organ systems, as well as radiation dose estimation of possible exposed individuals, is one of the key links to emergency medical assistance after radiation damage. The lung has raised significant concerns because of its radiosensitivity. Radiation-induced lung injury (RILI) has a serious impact on the quality of patients’ lives and limits the effect of radiotherapy (RT) for chest tumors, with 5–20% of patients experiencing this adverse effect [1]. The Clinical symptoms of radiation pneumonitis include a persistent dry cough, shortness of breath, mild fever or, occasionally, a high fever that may be secondary to radiation-induced pulmonary fibrosis and may even be the direct cause of death [2]. In the presence of extensive pulmonary fibrosis, antibiotic and corticosteroid therapeutics are limited, and there is no effective clinical treatment, which severely affects the patient’s quality of life and even their survival [3]. Macrophages, fibroblasts and T lymphocytes, as well as other inflammatory and immune cells, have key roles in the development of RILI. In addition, TGF-β, IL-4, IL-13 and IFN-γ have also been implicated in this process [4,5]. However, specific biological mechanisms and effective drug interventions for RILI remain to be fully elucidated. Current diagnosis methods for RILI, such as clinical biochemical indicators, lung function and medical imaging, have the drawbacks of sensitivity, specificity and lag effects [6]. Thus, identifying biomarkers for early diagnosis and revealing the molecular mechanisms of RILI is crucial for preventing disease progression, reducing patient mortality and taking effective measures as early as possible.

As an important component of systems biology, metabolomics is a comprehensive analysis of small-molecule metabolites and may reflect pathophysiologic states [7]. Metabolomics technologies have been developed over the past two decades to enable reliable identification, detection and quantification of novel metabolites in food, plant, environmental, animal and human studies [8] and have been widely adopted as a new approach for biomarker discovery and comprehensive understanding of the underlying pathogenesis [9]. To meet the demands of rapid radiation damage assessment in large-scale nuclear accidents, metabolomics has been attempted to identify biomarkers of radiation injury in various biological samples. Although the majority of studies have focused on a variety of biofluids derived from animal models (like mice, rats and non-human primates), as well as humans, the overlap in biomarkers of radiation injury across species has highlighted the metabolic pathways that are most perturbed, including β-oxidation of fatty acids (acylcarnitines), energy metabolism (TCA cycle intermediates), purines and pyrimidines metabolism, pro-inflammatory pathways (the omega-6 constituents, polyunsaturated fatty acids) and amino acids metabolism [10,11,12,13,14]. In the case of RILI, metabolomics has been utilized to reveal the metabolic characteristics of RILI in different genotypes of mice [15] and to explore the metabolic changes in serum and lung tissues exposed to irradiation [16,17]. These studies, however, primarily focus on the metabolic changes at a single time point post irradiation, which reflect the metabolic characteristics at a certain stage of RILI development and ignore the influence of time. In contrast, dynamic metabolomics could capture the variation generated by time and truly reveal dynamic metabolic changes during the development of RILI. Therefore, the combination of static and dynamic analyses is necessary to obtain the key metabolic characteristics related to RILI and discover the pathology of RILI.

Biofluid samples, such as plasma, serum, urine and saliva, are common sample types due to their convenient and minimally invasive collection. On the other hand, most biomarkers in biofluid samples only reflect the overall metabolic changes and cannot reflect the pathophysiologic change in injured tissues. In metabolomic studies of biofluid samples, unwanted confounding factors unrelated to diseases may lead to the discovery of false positive biomarkers [18]. For example, the metabolic characteristics of blood and urine are heavily influenced by gender, lifestyle, diet and other factors [19,20], which are difficult to unify. Therefore, metabolites found in biofluid samples are sometimes unable to accurately reflect the pathological status of disease. Nevertheless, tissue metabolomics can provide more abundant physiological or pathological information, which is important for diagnosis and treatment. Therefore, it is of importance to conjointly analyze differential metabolites in plasma and lung tissues.

In this study, metabolomics signatures of lung tissues and serial plasma specimens within 5 days of exposure to WTI in a rat model were performed. Furthermore, a panel of potential minimally invasive plasma metabolic markers for RILI was selected, followed by the assessment of radiation injury. Our findings will throw light on the molecular mechanism and serve as a strategy to aid in discovering minimally invasive diagnosis markers for RILI.

## 2. Materials and Methods

### 2.1. Chemicals and Reagents

Mass-grade methanol and acetonitrile were from Fisher Chemical (Thermo Fisher Scientific, Boston, MA, USA). Ammonium bicarbonate and formic acid were purchased from Fluka (Dresden, Germany). The ultrapure water was prepared with a Milli Q purification system (Millipore, Burlington, MA, USA). The chemical standards for compound identification were obtained from Sigma-Aldrich (St. Louis, MO, USA), Adamas (Hong Kong, China) or JK Chemical Ltd. (Shanghai, China). The deuterium-labeled internal standards (ISs), including cholic acid-d4,chenodeoxycholic acid-d4, succinic acid-d4,L-leucine-d10, L-phenylalanine-d5, L-tryptophan-d5, L-citrulline-d4, acylcarnitine C10:0-d3 and acylcarnitine C10:0-d3 were from Cambridge Isotope Laboratories, and the natural lipid analogs, including palmitic acid-d3and 1stearic acid-d3,were supplied by Avanti Polar Lipids.

### 2.2. Animals, Irradiation and Sample Collection

Female Wistar rats (170–190 g) were obtained from the Shanghai SLAC Laboratory Animal Ltd. (Shanghai, China), which were randomized into control (*n* = 13) and irradiated cohorts (*n* = 33). Prior to treatment, these animals were allowed to acclimate to the facility for one week. Then, these animals were anesthetized with 100 mg/kg ketamine and 10 mg/kg xylazine. To develop a radiation-induced lung injury rat model, we used a small animal radiotherapy treatment plan (X-RAD SmART) system. Images acquired through cone beam computed tomography (CT) were used to reconstruct and delineate targets. Multi-beam and CT-guided Monte Carlo-based plans were performed to optimize doses to targets. The terminal dose of WTI that the rats received was equivalent to either 10 Gy (*n* = 10), 20 Gy (*n* = 11) or 35 Gy (*n* = 12) at a dose rate of 2.7 Gy/min.

Plasma was obtained through periorbital bleeding at time points of 1, 2, 3 and 5 days post-irradiation, while lung tissue was collected on the fifth day post-irradiation. As some lung tissue samples have been exhausted for other analysis, a total of 39 samples (11 for controls, 10 for 10 Gy, 9 for 20 Gy, 9 for 35 Gy) were available from the cohort that was dedicated to the metabolomics study. All of the plasma and lung tissues were stored at −80 °C before LC-MS analysis.

The study was approved by the Ethics Committee of Soochow University.

### 2.3. Histology

Lung tissues from each group were immersed in 10% neutral buffered formalin and allowed to fix for a minimum of 24 h. The fixed lung specimens were embedded in paraffin, sliced into 4 μm thick sections and stained with hematoxylin and eosin (H&E) for analysis of tissue morphology changes following WTI.

### 2.4. LC-MS Pseudotargeted Metabolomics Analysis

To obtain more comprehensive metabolic characteristics, pseudotargeted metabolomics analyses based on LC-MS were used to determine lung or plasma metabolites. Meanwhile, considering that only known compounds can be biologically explained, the identified metabolites in the samples were kept for the metabolic analysis.

The composition and concentration of internal standards (ISs) for plasma and lung tissue are listed in Tables S1 and S2.

Plasma preparation: 200 μL ISs was added into 50 μL of sample for the protein precipitation. After vortexing, the sample was centrifuged at 13,000 rpm/min for 10 min (4 °C); the supernatant was taken and divided into two parts and dried by vacuum. Before LC-MS analysis, two dried supernatants were redissolved with 50 μL ACN: H_2_O (1:3, *v*/*v*).

Lung tissue preparation: About 20 mg of lung tissue samples were homogenized with ceramic beads in 1.5 mL ISs solution two times using a Tissue Lyser homogenizer (Gene Ready Ultracool, Life Real, Hangzhou, China). The homogenization took 5 min with 15 s intervals each time (45 HZ). Then, the homogenized tissue sample was centrifuged at 13,000 rpm/min for 15 min (4 °C), the supernatant was taken and divided into two parts, and dried by vacuum. Before LC-MS analysis, two dried supernatants were redissolved with 50 μL ACN: H_2_O (1:3, *v*/*v*). Before LC-MS analysis, two reconstituted samples were used for positive ion mode and negative ion mode, respectively.

Pseudotargeted analysis of plasma and lung tissue metabolites was performed on TSQ Vantage HPLC-MS/MS (Thermo Fisher, USA) with ESI, which was developed according to the proposed strategy described by Zheng et al. [21]. In the positive ion mode, a BEH C8 100 × 2.1 mm column (1.7 μm particle size, Waters) was employed for the separation. Mobile phase A was 0.1% aqueous formic acid in water. Mobile phase B consisted of 0.1% formic acid in acetonitrile. The linear gradient elution was set: 0–0.5 min, 5% B; 0.5–24 min, 5–100% B; 24–28 min, 100% B; 28–28.5 min, 100% B back to 5% B; 28.5–31.5 min, 5% B. An HSS T3 100 × 2.1 mm column (1.8 μm particle size, Waters) was utilized in the negative ion mode for the separation. The mobile phases were composed of 6.5 mM ammonium bicarbonate in water (C) and 6.5 mM ammonium bicarbonate in 95% methanol/water (D). The linear gradient elution followed: 0–1 min, 2% B; 1–20 min, 2–100% B; 20–24 min, 100% B; 24–24.5 min, 100% B back to 2% B; 24.5–27.5 min, 2% B. The flow rate was 0.25 mL/min in both positive and negative ion modes. The column temperature was kept at 50 °C, and the sample injection volume was 5 μL. The mass parameters with electrospray ionization were set as follows: 350 °C capillary temperature, 300 °C vaporize temperature, 35 arbitrary unit sheath gas flow rate, 10 arbitrary unit auxiliary gas flow rate, 3.0 kV capillary voltage for ESI+ mode and −2.5 kV for ESI mode.

### 2.5. Urea Detection

Urea contents were detected by Urea (BUN) Colorimetric Assay Kit (Urease Method). Samples and working reagent were added to the 96-well plate, and then absorbance at 580 nm was measured by microplate reader (BioTek, Winooski, VT, USA).

### 2.6. Quantitative Real-Time Polymerase Chain Reaction (q-RT-PCR)

Total RNA of the lung samples of SD rats were homogenized and isolated using RNA-Quick Purification Kit (ES Science, Shanghai, China), and cDNA synthesis was performed by the Reverse Transcription Reagent Kit (ABM, Vancouver, BC, Canada) according to the specification. Vii7 PCR system and SYBR^®^ Green PCR kit (QIAGEN, Hilden, Germany) were used for quantitative Real-Time Chain Reaction (q-RT-PCR). Data were normalized to the expression of α-tubulin in each sample. The forward and reverse primers used for qPCR were as follows:

CPT1A (Forward: 5’-CCTACCACGGCTGGATGTTT-3’, Reverse: 5’-TACAACATGGGCTTCCGACC-3’); CPT1B (Forward: 5’-ACAGGCATAAGGGGTGGCAT-3’, Reverse: 5’-CACTCCAATCCCACCTCGACC-3’).

### 2.7. Data Processing and Statistical Analysis

The integration of the peaks from pseudotargeted analysis was conducted by Xcalibur (LC-MS/MS). The metabolites with less than 20% missing values and relative standard deviation (RSD) below 30% in QC samples remained. Then, the peak area of the metabolite was normalized to ISs (for plasma) or ISs and tissue weight (for lung tissue), which was utilized for following data processing. A paired analysis with nonparametric test (two-tailed Wilcoxon signed-rank test) was performed to discover the differential features (*p* < 0.05) using the SPSS 16.0 software. False-discovery rate (FDR < 0.2) was used to reduce false-discovery rate. Heat map employing MeV 4.9.0 was used to visualize the metabolic regulations of the differential metabolites associated with ionizing radiation exposure.

The panel of potential biomarkers for radiation exposure was further refined by variable importance in projection (VIP) of partial least squares–discriminant analysis (PLS-DA). Multivariate statistical models, including principal component analysis (PCA), partial least-squares discriminant analysis (PLS-DA) and nonlinear kernel partial least squares (KPLS) combined with a preprocessing technique of orthogonal signal correction (OSC) [22], were carried out using SIMCA-P 11.5 demo version (Umetrics AB, Umeå, Sweden). Response permutation test with 200 times was conducted to assess whether the model was overfitting.

### 2.8. Metabolic Correlation Network Analysis

Metabolic correlation network was performed using Cytoscape software (version 2.8.3). In the correlation network map, the nodes represent the metabolites. The solid black and red edge lines show positive and negative relationships, respectively. Metabolites with Pearson correlation coefficients above a threshold (r ≥ 0.7, *p* < 0.05) were connected by lines.

## 3. Results

### 3.1. Histological Destruction of Rat Lung Tissues in Response to WTI

**Figure 1:**
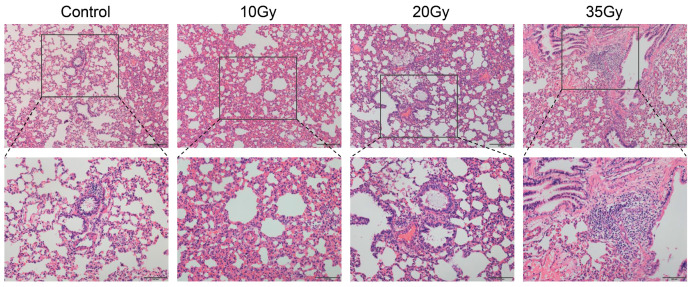
Representative images of HE staining in the control group and irradiated groups (first row: 40×, second row: 100×).

The hematoxylin–eosin (H&E) staining results showed that the structure of the lung tissue had severe destruction after exposure to radiation. As shown in Figure 1, the control group had a regular alveoli structure of lung tissue with slender alveoli and blood vessel walls. Compared with the control group, there was more diffuse hyperemia in the lung tissue at 5 d after 10 Gy irradiation. In the 20 Gy group, more inflammatory cells in the alveolar wall, thickening of the blood vessel wall and increased exudate in the alveoli could be observed. When it comes to 35 Gy, the lung tissue structure became more disorganized, with significant aggregation of lymphocytes, which reflected dose-dependent damage in lung tissues.

### 3.2. Ratlung Metabolic Signatures Exposed to WTI

To evaluate the stability of the analytical systems, quality control (QC) samples were evenly inserted into the analytical queue during the run of samples in LC-MS metabolomics analysis. QC samples were prepared similarly to the other samples. As shown in Figure S1A, the RSDs of 82.76, 94.58 and 99.01% of metabolites detected in QC samples were less than 10, 20 and 30%, respectively. In addition, QC samples were all within two times the standard deviation (SD) (Figure S1B). All of these results confirmed the reproducibility and stability of the metabolic profiling in LC-MS.

**Figure 2:**
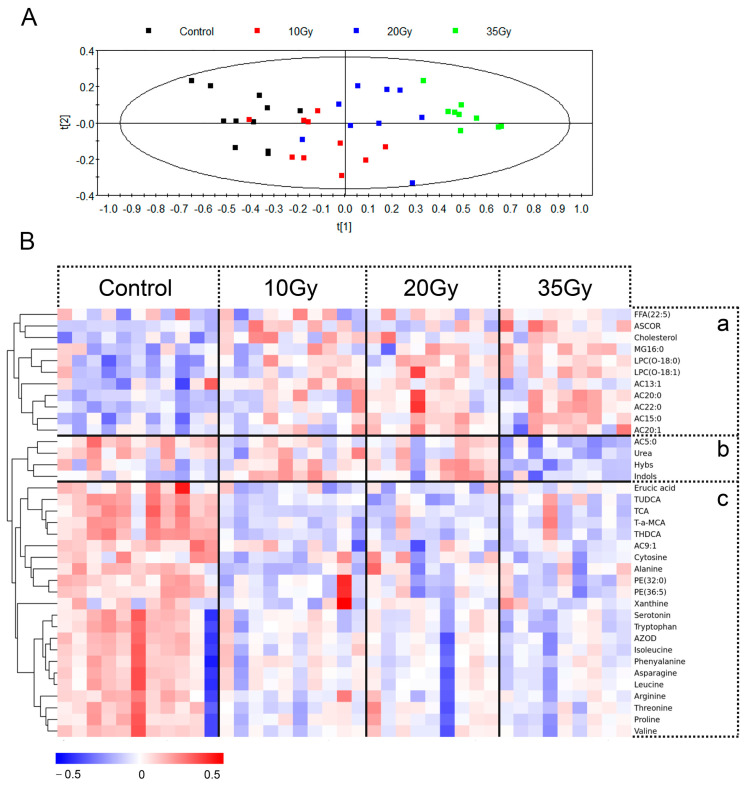
Statistical analysis for the data set of lung tissues at 5 d after WTI. (**A**) PLS-DA score plot comparing control and individual WTI doses. (**B**) Heatmap of the 37 differential metabolites in lung tissues, with the degree of changes compared with control group marked with colors. ASCOR: ascorbic acid; MG16:0: palmitoylglycerol; AC13:1: acylcarnitine C13:1; AC20:0: acylcarnitine C20:0; AC22:0: acylcarnitine C22:0; AC15:0: acylcarnitine C15:0; AC20:1: acylcarnitine C20:1; AC5:0: acylcarnitine C5:0; Hybs: 4−Hydroxybenzenesulfonic acid; Indols: indoxyl sulfate; TUDCA: tauroursodeoxycholic acid; TCA: taurocholic acid; T−α−MCA: tauro−α−Muricholic acid; AZOD: 3−Amino−2−oxazolidinone. The red and blue colors represent significant increases and decreases in response to WTI. According to the clustering, the metabolic alternations induced by WTI could be divided into three zones (a, b, c).

To highlight the separation of the study groups, a PLS-DA model was used to perform a multivariate pattern recognition analysis, and two principal components (PCs) were calculated based on this PLS-DA model. As shown in Figure 2A, except for a lesser overlap between the 10 and 20 Gy irradiated groups, there was a clear clustering trend and dose-dependent distance in different groups, which indicated that metabolic disorders in lung tissues were positively related to radiation doses.

Based on the analysis of variance *p*-value (ANOVA, *p* < 0.05) and false-discovery rate (FDR < 0.2), 37 differential metabolites associated with RILI were selected; the effects of irradiation on the differential metabolites are listed in Table S3 and visualized in Figure 2B. The metabolic alternations induced by WTI could be divided into three zones (a, b, c) according to the clustering. Metabolites in zone A were significantly up-regulated following WTI irradiation, mainly including lipid metabolites and fatty acid β-oxidation-related metabolites, such as cholesterol, lysophosphatidylcholine and long-chain AC. Meanwhile, most metabolites in panel C (bile acid and lipid metabolites) showed a significant decrease following irradiation. Additionally, metabolites in region B, containing acylcarnitine C5:0 (AC5:0), urea, 4-Hydroxybenzenesulfonic acid (Hybs) and indoxyl sulfate (Indols) were only down-regulated in the 35 Gy group.

### 3.3. Plasma Metabolic Signatures Exposed to WTI

Similarly, we assess the stability of the total metabolic profiling analytical systems in plasma. The RSDs of 43.60%, 83.72% and 95.93% of metabolites were less than 10%, 20% and 30%, respectively (Figure S2A), and QC samples were also all within two times the SD (Figure S2B). These results confirmed the reliability of metabolic profiling in LC-MS.

**Figure 3:**
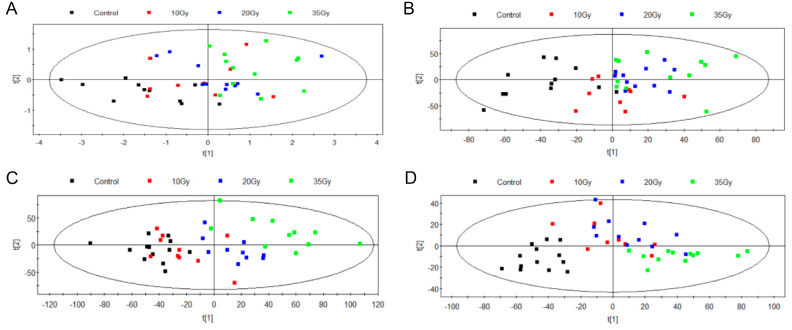
Statistical analysis for the data set in plasma at different times after WTI. PLS−DA score plot comparing control and individual WTI doses ((**A**), 1 d; (**B**), 2 d; (**C**), 3 d; (**D**), 5 d).

The plasma metabolomic profiles from 1 d to 5 d after were subsequently depicted on the basis of the PLS-DA model (Figure 3). There were two types of metabolic derangements, including radiation-induced changes and time-associated changes. Individual doses shown in Figure 3A could not be distinguished at 1 d after WTI, reflecting the lack of sensitivity of differential metabolites to identify the WTI doses. Differently from 1 d, the 2 d PLS-DA plots could distinguish the control and irradiated groups, although different WTI doses could not be clearly divided (Figure 3B). At 3 d after WTI, individual doses could be distinguished clearly, whereas the control group overlapped with the 10 Gy group (Figure 3C). Compared with 3 d, PLS-DA plots at 5 d after WTI showed that the control group clustered closely apart from the irradiated groups, and the high-WTI dose (35 Gy) group dispersed from the moderate-WTI dose (10 and 20 Gy) groups (Figure 3D). It is clear that the distance between the control area and the irradiated group became farther with the extension of time and increase in radiation exposure doses. These results suggested that irradiation could lead to metabolic disorders, and the degree of disorders in plasma was also positively related to radiation doses. Finally, there were 40, 84, 109 and 128 differential metabolites reaching significance in plasma selected at 1 d, 2 d, 3 d and 5 d after irradiation, respectively (Tables S4–S7).

### 3.4. Potential Plasma Metabolite Markers of Radiation-Induced Lung Injury

To select a panel of biomarkers for clinical RILI diagnosis and prognosis, metabolic data in plasma and lung tissues were analyzed conjointly. We initially selected 37 and 123 differential metabolites in lung tissue and plasma, respectively. Among these metabolites, 23 metabolites exhibited significant changes in both comparison groups simultaneously (Figure S3A).

In order to reduce the risk of false positives, checking the metabolites before identifying the biomarker candidates in this discovery phase is necessary. The first step was to construct the PLS model based on the intersection of 23 metabolites and analyze the variable importance (VIP) of each metabolite. We used two principal components (PCs) in plasma and lung tissues to screen differential metabolites. As shown in Figure S3B, 10 metabolites were obtained to preserve the statistical importance of the classification in two PCs based on VIP (VIP > 1). Secondly, a correlation analysis was performed on the above 10 metabolites to select metabolites with high correlation in plasma and lung tissue, and all these metabolites were obtained based on the correlation (Table S8). Finally, the VIP values of the 10 elected metabolites were analyzed again to assess the contribution to classification. Then, the top seven metabolites (taurocholic acid (TCA), acylcarnitine C5:0 (AC5:0), Leucine, tauroursodeoxycholic acid (THDCA), tauro-α-Muricholic acid (T-α-MCA), acylcarnitine C9:1 (AC9:1) and urea) with the highest VIP scores were selected as the most potential panel of plasma metabolic makers for RILI (Figure S4).

Subsequently, the potential panel of metabolic markers was assessed by the OSC-KPLS model to discriminate different dose groups at different stages of radiation exposure. Figure S5 shows the clustering graph of the control and irradiated groups at 1 d, 2 d, 3 d and 5 d after WTI. Each data point represents a real sample, with the vertical coordinate representing the actual radiation dose received and the horizontal coordinate representing the injury classification. The comparison between the predicted radiation doses and the observed values based on the panel is displayed in Table S9. Table 1 shows the classification results at different time points after irradiation. Compared with the early stage (1 d, 2 d after WTI), nearly all predicted values were close to the observed values at a later stage (3 d, 5 d after WTI), with the accuracies of classification all more than 80%. The result indicated the potential of the panel for estimating the approximate radiation dose and being a biomarker of RILI.

In order to directly trace the changes of the potential metabolic markers in the early stages of RILI, we analyzed dynamic plasma metabolic data within 5 days post WTI. As shown in Figure S6, the majority of screened metabolites began to show a significant difference at 2 d after WTI, except urea. Urea showed a down-regulated trend from 1 d to 5 d. AC5:0 and AC9:1 began to decrease at 2 d and 3 d, respectively. Aminoacids (leucine) began to decrease at a later period (3 d after WTI). Cholic acid levels, including TCA, THDCA and T-a-MCA, began to show significant decreases until 5 d.

To further explore the temporal trajectory of these metabolites, the levels for each irradiated rat were divided by the average controls to rule out metabolic derangement due to time-related changes (Figure S7). Interestingly, despite the complexity of radiation regulations, most of them displayed a monotonic response in 20 Gy and 35 Gy-irradiated cohorts from 2 d to 5 d post-irradiation. These features are considered the key metabolites with consistent changing tendencies when comparing high doses versus low doses and low doses versus Pre, reflecting the temporal variations with RILI progression and indicating their potential for assessment of radiation injury for RILI.

### 3.5. Metabolic Correlation Network Analysis

Due to complex physicochemical reactions, not only did metabolite levels show significant changes, but linkages between metabolites could also be altered [23]. The correlation network could provide an overview of a given status of the complex biological system and reveal dysregulated biochemical mechanisms associated with the stimulus [24]. A positive correlation between two metabolites indicates the adjacent relationship in a metabolic pathway, whereas a negative correlation indicates that one of two metabolites is used to generate the other one directly or indirectly [25].

**Figure 4:**
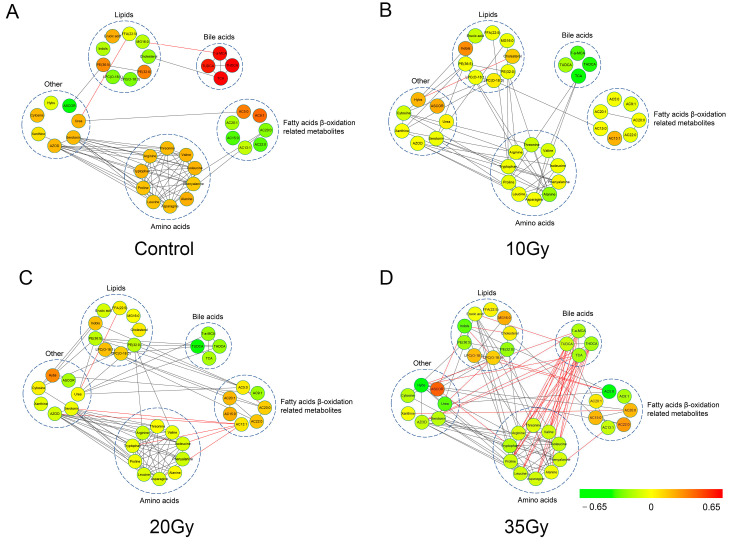
Metabolic correlation network analysis based on differential metabolites associated with radiation injury in the control and irradiated groups at 5 d after WTI ((**A**), Control; (**B**), 10 Gy; (**C**), 20 Gy; (**D**), 35 Gy). Nodes represent the metabolites, and the lines between nodes represent their relationship associated with biochemical reactions. ASCOR: ascorbic acid; MG16:0: palmitoylglycerol; AC13:1: acylcarnitine C13:1; AC20:0: acylcarnitine C20:0; AC22:0: acylcarnitine C22:0; AC15:0: acylcarnitine C15:0; AC20:1: acylcarnitine C20:1; AC5:0: acylcarnitine C5:0; Hybs: 4-Hydroxybenzenesulfonic acid; Indols: indoxyl sulfate; TUDCA: tauroursodeoxycholic acid; TCA: taurocholic acid; T-α-MCA: tauro-α-Muricholic acid; AZOD: 3-Amino-2-oxazolidinone. The metabolites (nodes) in red and green colors represent significant increases and decreases in response to WTI. The black and red lines between metabolites represent positive and negative relationships, respectively.

Then, the correlation network analysis between differential metabolites was performed to reveal the metabolic regulations following WTI using Cytoscape software. The linkage line of metabolites was based on the Pearson correlation coefficient by calculating the relative levels of the metabolites. As shown in Figure 4, most metabolites connected with each other and more connections between different metabolites could be observed after irradiation, which inferred the complex metabolic regulation in RILI.The metabolites with more connections to others may play a more important role in the metabolic regulations of radiation exposure.

Lipids were found to correlate more positively with amino acids with increased radiation exposure doses. In contrast, bile acids displayed a more negative correlation with amino acids, such as arginine, proline, leucine and asparagine, in the 35 Gy-irradiated group. In the 20 Gy irradiated group, fatty acid β-oxidation-related metabolites began to show strong correlations with lipids. β-oxidation is a process of generating energy by the formation of ketone bodies from fatty acids, which could explain the correlation between lipids and β-oxidation and indicate the significant contribution in the process of RILI. These results indicated the important roles of bile acids, lipids and fatty acid β-oxidation-related metabolites in the metabolic regulation of RILI.

### 3.6. CPT1 Gene Expression Level and Enzyme Activity in the Lung Samples of Rats Exposed to WTI

We further found that the levels of Acylcarnitine C20:1 and Acylcarnitine C20:1 increased at 1 d after exposure and maintained the change up to 5 d, indicating the radiosensitivity of fatty acid β-oxidation (Figure S8A). Thus, we evaluated the carnitine acyltransferases (CPT1, presenting in the mitochondrial outer membrane; CPT2, situating at the matrix side of the inner membrane) involved in acylcarnitine metabolism, which regulates this transport system [26]. The activities of two enzymes can be estimated by ratios, such as the CPT1 ratio (carnitine/(C16:1 + C18:0)) and the CPT2 ratio (C16:0 + C18:1/C2). An elevation of the CPT1 ratio indicates CPT1 deficiency or impaired functions [27], reflecting the increased mitochondrial entrance of long-chain FA. Meanwhile, the increase in the CPT2 ratio points to a significant reduction in long-chain fatty acid oxidation or impaired CPT2 functions, which means long-chain acylcarnitine cannot be converted to their corresponding acyl-CoA esters [26]. As can be seen in Table 2, the CPT1 ratio significantly decreased in response to WTI, while the CPT2 ratio markedly increased, indicating the accumulation of long-chain acylcarnitine in mitochondria.

In addition, to preliminarily discover the extra accumulation of long-chain acylcarnitine after WTI, the mRNA levels of CPT1A and CPT1B in lung tissues from model rats were analyzed at 5 d after WTI. As shown in Figure S8B, the mRNA levels of CPT1A and CPT1B showed significant dose-dependent elevation compared with the control group. This further substantiates that radiation plays a crucial role in the regulation of CPT1 activity, which may lead to the accumulation of long-chain acylcarnitine.

## 4. Discussion

In the current study, not only the metabolite levels but also the metabolic correlation networks were significantly altered following WTI. These metabolic abnormalities are mainly involved in amino acids, bile acids, lipids and fatty acid β-oxidation-related metabolites, which are discussed in the following sections.

### 4.1. Amino Acids

Amino acids are a kind of vital metabolite in the organism, the basic components of proteins and have biological functions such as synthesizing hormones, transmitting cell signals and regulating gene expression [28]. After WTI, the levels of amino acids, including arginine, phenylalanine, tryptophan, valine, leucine, isoleucine, threonine, proline and alanine were significantly reduced in lung tissues (Table S3). These amino acids are involved in multiple metabolic pathways, such as arginine and proline metabolism; valine, leucine and isoleucine degradation;the urea cycle; and aspartate metabolism.

As essential amino acids, branched-chain amino acids (BCAAs, leucine, isoleucine and valine) are widely studied due to their crucial role in the regulation of protein synthesis, primarily through the activation of the mTOR signaling pathway and their growing recognition as players in the regulation of a variety of physiological and metabolic processes [29,30]. Elevated blood BCAA levels in both animal models and humans following total body irradiation (TBI) have been implicated in radiation-induced activated protein breakdown [13,22,31,32]. In contrast to the above findings, BCAAs showed obvious reduced levels in both lung tissues and plasma in response to WTI (Figure S9). BCAAs are ketogenic and glycogenic amino acids, which can be converted to branched-chain keto acids (BCKAs) through BCAA transaminase (BCAT) in body metabolism [33]. Decreased blood BCAA levels have been reported in patients with chronic obstructive pulmonary disease (COPD), while dietary supplementation with BCAAs ameliorates COPD-related weight loss and respiratory muscle weakness [34,35,36,37,38]. It has been found that the inhibition of BCAT can inhibit airway inflammation and remodeling [39], suggesting that BCAT activity is related to pneumonia response. Furthermore, mTOR signaling is closely associated with the dysregulation of autophagy, inflammation, as well as cell growth and survival, resulting in the development of pulmonary fibrosis [40]. Studies suggest that mTOR inhibitors are promising modulators of radiation-induced pulmonary fibrosis (RIPF) [41]. Given that BCAAs have been recognized as having anabolic effects in protein metabolism, which involve the activation of the mTOR pathway, lower BCAAs levels may cause the dysregulation of the mTOR pathway, leading to RILI.Therefore, identifying the BCAA metabolic pathway may be a potential attractive treatment for therapeutic targets in RILI.

Decreases in urea and arginine in lung tissues indicate a urea cycle disorder following WTI. Arginine engages in the urea cycle in the body, promoting the formation of urea, thus transforming ammonia in the human body into non-toxic urea and reducing blood ammonia concentrations [42]. Ionizing radiation could cause the urea-to-ammonia ratio to drop precipitously and thus give rise to hyperammonemia [43,44], suggesting the disturbance of the urea cycle and agreeing with our findings with declined levels of pulmonary arginine and urea. Arginine is also involved in the nitric oxide (NO) pathway and is a substrate for the synthesis of endogenous NO catalyzed by the enzyme NO synthase (NOS) [45]. Elevated exhaled NO following thoracic radiation has been reported to be predictive of RILI. Recent studies have shown that arginine has an important protective effect on pulmonary inflammation and fibrosis [46,47,48]. Moreover, the supplementation of arginine can significantly downregulate procollagen mRNA transcription and hydroxyproline content in lung tissues [49]. Consequently, we conclude that the decline of pulmonary arginine may be related to the injury repair of the body in response to WTI, which further results in the depletion of proline.

Phenylalanine and tryptophan belong to aromatic amino acids, which can synthesize acute proteins in response to inflammation [50]. Such proteins can play an anti-inflammatory effect through immune regulation [51]. Therefore, the disturbance of aromatic amino acid metabolism in WTI rats may be related to the immune response of RILI. In addition, tryptophan is the only precursor of serotonin, which is a key monoamine neurotransmitter that participates in the modulation of central neurotransmission and physiological function in the enteric system [52]. In addition, tryptophan can be metabolized to kynurenine, tryptamine and indole, modulating neuroendocrine and gut immune responses [52]. In our study, phenylalanine, tryptophan and serotonin were significantly decreased in lung tissue in response to WTI, while indoxyl sulfate (Indols) was increased at low doses (10 Gy, 20 Gy) and decreased at the high dose (35 Gy) (Figure S10). The changed levels of all these metabolites in lung tissues implied a role for gut microflora in the lung tissue exposed to WTI. Chen et al. reported that fecal microbiota transplantation (FMT) attenuated radiation pneumonia, scavenged oxidative stress and ameliorated lung function in mouse models following local chest irradiation [53]. This research further indicates the relationship between radiation pneumonia and intestinal flora metabolism, which is consistent with the results of this study. Furthermore, the decreased levels of plasma metabolites associated with tryptophan, including kynurenic acid, serotonin and indole at 3–5 d post-irradiation, reinforced the importance of gut microflora in RILI.

### 4.2. Bile Acids

Bile acids (BAs) are synthesized in the liver and secreted into the digestive tract, where they facilitate the digestion and absorption of lipids. They are associated with chronic inflammation and remodeling of the lung microbiota. Furthermore, BAs can regulate the composition of the microbiota in indirect or direct ways and protect the gut barrier [54]. In recent years, increasing evidence has demonstrated that there is a close connection between gut microbes and the lung by modulating the transmission route of the gut–lung axis [55]. Many lung diseases often present with dysbiosis of the gut flora, which may refer to the development of disease [54]. Additionally, BAs participate in the interactions between the intestinal microbiota and the host’s immunity [56]. It has been shown that BAs can act as signaling molecules via the activation of dedicated receptors, such as nuclear receptor Farnesoid X Receptor (FXR) and membrane-bound receptor Takeda-G protein receptor 5 (TGR5). In addition, the FXR for BAs has been shown to be expressed in human airway epithelial cells [57], and the agonists have been proven to have beneficial effects in a wide range of pulmonary diseases, such as chronic obstructive pulmonary disease (COPD) and idiopathic pulmonary fibrosis [58]. Recent reports verified the therapeutic effects of the natural agonists of FXR (DCA and LCA) on inflammatory bowel disease by restoring intestinal barrier function and alleviating inflammatory reactions [59]. In our research, taurocholic acid (TCA), taurohyodeoxycholic acid (THDCA), Taurohyodeoxycholic acid (TUDCA) and tauro-α-Muricholic acid (T-α-MCA) were significantly decreased in the lung tissues of rats in response to WTI, which revealed that RILI induced the disturbance of bile acid metabolism and gut barrier dysfunction. These results are in agreement with a recent study by Li et al., who reported that the bile acid pool had a marked reduction after whole chest irradiation and was recovered bycryptotanshinone (CPT) treatment in large part [55].

### 4.3. Lipids and Fatty Acid β-Oxidation

It is well known that lipids are the major constituents of cell membrane bilayers, playing a major role in cell signaling, membrane anchorage and substrate transport.Radiation exposure causes dysfunction of the cell membrane and disrupted lipid metabolism, together with changes in lipid concentration and increased lipid peroxidation [60,61]. Increasing studies have indicated that irradiation resulted in lipid accumulation, evidenced by elevated triacylglycerol and cholesterol levels in plasma, liver or lung tissues [12,62,63,64]. In accordance with the lipid accumulation revealed in the above reports, pulmonary palmitoylglycerol, cholesterol, unsaturated free fatty acid 22:5 (FFA 22:5), LPC (LPC(O-18:0) and LPC(O-18:1)) were significantly increased in response to WTI (Figure S11). Although the exact mechanism of radiation alters lipid mechanism is unclear, increased glucose catabolism by providing increased levels of glycerophosphate as a lipid precursor and up-regulated the lipoprotein lipase and fatty acid binding protein expression have been considered important contributors to this lipid accumulation [65,66]. Moreover, pulmonary lipid metabolites could induce chronic inflammation in tissues primarily by promoting the infiltration and activation of macrophages [4]. Among the affected lipids, the alterations of two PEs, including PE 32:0 and PE 36:5, deserve attention (Figure S11). As the second most abundant membrane phospholipid in mammals, PE plays an essential role in mammalian development and cellular processes, including metabolism and signaling [67]. It has been demonstrated that PE strongly contributes to surfactant-induced inhibition of collagen expression in human lung fibroblasts via a Ca^2+^ signal, and early administration of PE-enriched Beractant decreases lung fibrosis in mice [68]. Thus, decreased levels of pulmonary PEs in the irradiated groups may be indicative of injury repair in RILI at the expense of consumption. Overall, these findings support the idea that an alteration in lipid metabolism is important in RILI pathology.

The β-oxidation of fatty acids is one of the main methods of energy metabolism in organisms [69], including the activation, transfer and oxidation of fatty acids, culminating in the production of acetyl-CoA and direct involvement in the tricarboxylic acid cycle (TCA) or generation of metabolites, such as ketone bodies for energy metabolism. Acylcarnitines (ACs) are formed when fatty acid enters the mitochondria for β-oxidation through the carnitine shuttle. ACs can be divided into short (C3–C5), medium (C6–C12) and long-chain (>C12) ACs depending on the length of the acyl groups. Due to the large number and special structure, ACs play an important role in the physiological activities of cells and become a key substance for cellular metabolism [70]. Levels of ACs can vary depending on the metabolic conditions but may accumulate when rates of β-oxidation exceed those of tricarboxylic acid cycle (TCA). ACs play a major role in the β-oxidation of long-chain fatty acids (LCFAs) and serve as a carrier to transport activated long-chain acyl-CoAs into the mitochondria for subsequent β-oxidation to provide energy for cellular activities [71].

Prior studies have implicated carnitine metabolites as potential biomarkers of radiation injury in biofluids derived from animals and humans [10,11,72]. Meanwhile, enhancement of AC levels has been reported in the small intestine of abdominal-irradiated rats [73]. In the current study, the decreased CPT1 ratio and increased CPT2 ratio in response to WTI (Table 2) reflected increased mitochondrial entrance of long-chain ACs and incomplete fatty acid β-oxidation, accounting for the accumulation of long-chain ACs in both lung tissues and plasma (Tables S3–S7).

## 5. Conclusions

In the present study, early time-point plasma and lung metabolic signatures following WTI were revealed. To identify minimally invasive markers for RILI, the metabolic features of the lung tissue in response to WTI were cross-correlated with plasma metabolic features. In the combined multivariate PLS model, the panel of potential plasma metabolic markers was selected and used to assess the radiation injury levels within 5 days following WTI. Our data implied that plasma metabolites can potentially be used to estimate radiation doses associated with RILI. Moreover, the significant difference in metabolite levels and metabolic correlation network in the lung tissue revealed that multiple metabolic dysregulation primarily involved amino acids, bile acids, lipids and fatty acid β-oxidation-related metabolites. In particular, the accumulation of long-chain ACs deserves attention by jointly analyzing dynamic plasma metabolic characteristics. These findings provide insight into the metabolic characteristics associated with RILI. Further extensive studies, including the validation of the panel of potential metabolic markers for RILI by another cohort of animals and thoracic radiotherapy patients and exploration of the effect of fatty acid β-oxidation metabolism in RILI, are required.

## Figures and Tables

**Table 1:** Classification of radiation injury at different time points after WTI based on OSC-KPLS model and the panel of potential biomarkers.

Triage	Control	Mild	Moderate	Severe
Accuracy of classification at 1 d	72.7%	90.0%	58.3%	91.7%
Accuracy of classification at 2 d	92.3%	50.0%	72.7%	66.7%
Accuracy of classification at 3 d	100.0%	90.0%	100.0%	100.0%
Accuracy of classification at 5 d	100.0%	80.0%	81.8%	100.0%

**Table 2:** Related ratios in control, 10 Gy, 20 Gy and 35 Gy groups.

Enzymes	Control	10 Gy	20 Gy	35 Gy
CPT1	155.4 (139.5–197.4)	129.4 * (109.6–140.2)	112.4 ** (100.2–128.7)	104.1 ** (94.9–112.3)
CPT2	0.004 (0.003–0.004)	0.005 * (0.004–0.005)	0.005 ** (0.005–0.006)	0.006 ***^,#^ (0.005–0.006)

Note: Values are expressed as medians (25th, 75th percentiles). The *p*-values were calculated based on nonparametric Kruskal–Wallis test. Compared with control, * *p* < 0.05, ** *p* < 0.01, *** *p* < 0.001; compared with 10 Gy, ^#^ *p* < 0.05.

## Data Availability

All data that support the findings of the study are within the manuscript or in Supplemental Materials.

## Supplementary Materials

The following supporting information can be downloaded at: https://www.mdpi.com/article/10.3390/metabo13091020/s1, Figure S1: Reproducibility and stability of metabolic profiling in lung tissues were assessed. (A) RSD distribution of the QC samples. (B) PCA score plots of the QC sample distributions in LC-MS; Figure S2: Reproducibility and stability of metabolic profiling in plasma. (A) RSD distribution of the QC samples. (B) PCA score plots of the QC sample distributions in LC-MS; Figure S3: Screen of biomarkers in plasma. (A) Venn diagram showing the shared and unique metabolites between plasma and lung tissues. (B–E) Column plots of VIP value in plasma and lung tissues, metabolites with VIP [1] and VIP [2] > 1 were selected; Figure S4: Column plots of VIP value in plasma; metabolites with more importance were selected as biomarkers; Figure S5: (A,C,E,G) Comparison between the predicted radiation doses and observed values in plasma at 1 d, 2 d, 3 d and 5 d after WTI. (B,D,F,H) Validation plots obtained from 200 permutation tests based on the panel of these 7 biomarkers, and the model parameters showed the predictive ability of model; Figure S6: Dynamic changes of these 7 potential biomarkers in plasma.Compared with Control, * *p* < 0.05, ** *p* < 0.01; Figure S7: Dynamic changes of these 7 potential biomarkers in plasma (normalized by control group); Figure S8: (A,B) Dynamic changes of Acylcarnitine C20:0 and Acylcarnitine C20:1 in plasma (normalized by control group). (C,D) The mRNA level of CPT1A and CPT1B in lung tissues at 5 d after different radiation dose exposures.Compared with Control, * *p* < 0.05, ** *p* < 0.01, *** *p* < 0.001; Figure S9: (A,B) Dynamic changes of valine and leucine in plasma. (C–E) Changes of valine, leucine and isoleucine in lung tissues. Compared with Control, * *p* < 0.05, ** *p* < 0.01; Figure S10: (A,B) Changes in aromatic amino acids (phenylalanine and tryptophan) in lung tissues. (C,D) Changes intryptophan-related metabolites (serotonin and indoxyl sulfate) in lung tissues.Compared with Control, * *p* < 0.05, ** *p* < 0.01; compared with 10 Gy, ^##^ *p* < 0.01; compared with 20 Gy, ^&&^ *p* < 0.01; Figure S11: Changes in lipids (LPC (0–18:0, LPC (O-18:1), PE (32:0) and PE (36:5)) in lung tissues.Compared with control, * *p* < 0.05, ** *p* < 0.01; Table S1: Concentration of internal target used for pretreatment of plasma samples; Table S2: Concentration of internal target used for pretreatment of lung tissue samples; Table S3: Statistical information of differential metabolites in lung tissues at 5 d after WTI (X ± SD); Table S4: Statistical information of differential metabolites in plasma at 1 d after WTI (X ± SD); Table S5: Statistical information of differential metabolites in plasma at 2 d after WTI (X ± SD); Table S6: Statistical information of differential metabolites in plasma at 3 d after WTI (X ± SD); Table S7: Statistical information of differential metabolites in plasma at 5 d after WTI (X ± SD); Table S8: Correlation analysis of differential metabolites in plasma and lung tissues at 5 d after WTI; Table S9: Stimulation of RILI classification after WTI.

## References

[1] Hanania A.N., Mainwaring W., Ghebre Y.T., Hanania N.A., Ludwig M. (2019). Radiation-Induced Lung Injury: Assessment and Management. Chest.

[2] Inoue A., Kunitoh H., Sekine I., Sumi M., Tokuuye K., Saijo N. (2001). Radiation pneumonitis in lung cancer patients: A retrospective study of risk factors and the long-term prognosis. Int. J. Radiat. Oncol. Biol. Phys..

[3] Liu X., Shao C., Fu J. (2021). Promising Biomarkers of Radiation-Induced Lung Injury: A Review. Biomedicines.

[4] Sunil Gowda N.S., Raviraj R., Nagarajan D., Zhao W. (2019). Radiation-induced lung injury: Impact on macrophage dysregulation and lipid alteration—A review. Immunopharmacol. Immunotoxicol..

[5] Yan Y., Fu J., Kowalchuk R.O., Wright C.M., Zhang R., Li X., Xu Y. (2022). Exploration of radiation-induced lung injury, from mechanism to treatment: A narrative review. Transl. Lung Cancer Res..

[6] Zhang T., Zhou Z., Wen L., Shan C., Lai M., Liao J., Zeng X., Yan G., Cai L., Zhou M. (2023). Gene Signatures for Latent Radiation-Induced Lung Injury Post X-ray Exposure in Mouse. Dose Response.

[7] Liu R., Bao Z.X., Zhao P.J., Li G.H. (2021). Advances in the Study of Metabolomics and Metabolites in Some Species Interactions. Molecules.

[8] Di Minno A., Gelzo M., Caterino M., Costanzo M., Ruoppolo M., Castaldo G. (2022). Challenges in Metabolomics-Based Tests, Biomarkers Revealed by Metabolomic Analysis, and the Promise of the Application of Metabolomics in Precision Medicine. Int. J. Mol. Sci..

[9] Trivedi D.K., Hollywood K.A., Goodacre R. (2017). Metabolomics for the masses: The future of metabolomics in a personalized world. NewHoriz. Transl. Med..

[10] Laiakis E.C., Mak T.D., Anizan S., Amundson S.A., Barker C.A., Wolden S.L., Brenner D.J., Fornace A.J. (2014). Development of a metabolomic radiation signature in urine from patients undergoing total body irradiation. Radiat. Res..

[11] Pannkuk E.L., Laiakis E.C., Authier S., Wong K., Fornace A.J. (2016). Targeted Metabolomics of Nonhuman Primate Serum after Exposure to Ionizing Radiation: Potential Tools for High-throughput Biodosimetry. RSC Adv..

[12] Feurgard C., Bayle D., Guezingar F., Serougne C., Mazur A., Lutton C., Aigueperse J., Gourmelon P., Mathe D. (1998). Effects of ionizing radiation (neutrons/gamma rays) on plasma lipids and lipoproteins in rats. Radiat. Res..

[13] Hong X., Tian L., Wu Q., Gu L., Wang W., Wu H., Zhao M., Wu X., Wang C. (2023). Plasma metabolomic signatures from patients following high-dose total body irradiation. Mol. Omics.

[14] Pannkuk E.L., Fornace A.J., Laiakis E.C. (2017). Metabolomic applications in radiation biodosimetry: Exploring radiation effects through small molecules. Int. J. Radiat. Biol..

[15] Stirling E.R., Cook K.L., Roberts D.D., Soto-Pantoja D.R. (2019). Metabolomic Analysis Reveals Unique Biochemical Signatures Associated with Protection from Radiation Induced Lung Injury by Lack of cd47 Receptor Gene Expression. Metabolites.

[16] Gao Y., Li X., Gao J., Zhang Z., Feng Y., Nie J., Zhu W., Zhang S., Cao J. (2019). Metabolomic Analysis of Radiation-Induced Lung Injury in Rats: The Potential Radioprotective Role of Taurine. Dose Response.

[17] Feng Y., Gao Y., Tu W., Feng Y., Cao J., Zhang S. (2022). Serum Metabolomic Analysis of Radiation-Induced Lung Injury in Rats. Dose Response.

[18] Li Y., Li M., Jia W., Ni Y., Chen T. (2018). MCEE: A data preprocessing approach for metabolic confounding effect elimination. Anal. Bioanal. Chem..

[19] Slupsky C.M., Rankin K.N., Wagner J., Fu H., Chang D., Weljie A.M., Saude E.J., Lix B., Adamko D.J., Shah S. (2007). Investigations of the effects of gender, diurnal variation, and age in human urinary metabolomic profiles. Anal. Chem..

[20] Cross A.J., Moore S.C., Boca S., Huang W.Y., Xiong X., Stolzenberg-Solomon R., Sinha R., Sampson J.N. (2014). A prospective study of serum metabolites and colorectal cancer risk. Cancer.

[21] Zheng F., Zhao X., Zeng Z., Wang L., Lv W., Wang Q., Xu G. (2020). Development of a plasma pseudotargeted metabolomics method based on ultra-high-performance liquid chromatography-mass spectrometry. Nat. Protoc..

[22] Tang X.X., Zheng M.C., Zhang Y.Y., Fan S.J., Wang C. (2013). Estimation value of plasma amino acid target analysis to the acute radiation injury early triage in the rat model. Metabolomics.

[23] Yao X.T., Xu C., Cao Y.R., Lin L., Wu H.X., Wang C. (2019). Early metabolic characterization of brain tissues after whole body radiation based on gas chromatography-mass spectrometry in a rat model. Biomed. Chromatogr..

[24] Bonelli R., Woods S.M., Ansell B.R.E., Heeren T.F.C., Egan C.A., Khan K.N., Guymer R., Trombley J., Friedlander M., Bahlo M. (2020). Systemic lipid dysregulation is a risk factor for macular neurodegenerative disease. Sci. Rep-UK.

[25] Wang L., Hou E.T., Wang L.J., Wang Y.J., Yang L.J., Zheng X.H., Xie G.Q., Sun Q., Liang M.Y., Tian Z.M. (2015). Reconstruction and analysis of correlation networks based on GC-MS metabolomics data for young hypertensive men. Anal. Chim. Acta.

[26] Yang Y., Sadri H., Prehn C., Adamski J., Rehage J., Danicke S., Saremi B., Sauerwein H. (2019). Acylcarnitine profiles in serum and muscle of dairy cows receiving conjugated linoleic acids or a control fat supplement during early lactation. J. Dairy. Sci..

[27] Fingerhut R., Roschinger W., Muntau A.C., Dame T., Kreischer J., Arnecke R., Superti-Furga A., Troxler H., Liebl B., Olgemoller B. (2001). Hepatic carnitine palmitoyltransferase I deficiency: Acylcarnitine profiles in blood spots are highly specific. Clin. Chem..

[28] Wu G. (2009). Amino acids: Metabolism, functions, and nutrition. Amino Acids.

[29] Laplante M., Sabatini D.M. (2012). mTOR Signaling in Growth Control and Disease. Cell.

[30] Blomstrand E., Eliasson J., Karlsson H.K.R., Kohnke R. (2006). Branched-chain amino acids activate key enzymes in protein synthesis after physical exercise. J. Nutr..

[31] Holecek M., Skopec F., Sprongl L., Mraz J., Skalska H., Pecka M. (2002). Effect of alanyl-glutamine on leucine and protein metabolism in irradiated rats. Amino Acids.

[32] Khan A.R., Rana P., Devi M.M., Chaturvedi S., Javed S., Tripathi R.P., Khushu S. (2011). Nuclear magnetic resonance spectroscopy-based metabonomic investigation of biochemical effects in serum of gamma-irradiated mice. Int. J. Radiat. Biol..

[33] Holecek M. (2020). Why Are Branched-Chain Amino Acids Increased in Starvation and Diabetes?. Nutrients.

[34] Crowley G., Kwon S., Haider S.H., Caraher E.J., Lam R., St-Jules D.E., Liu M., Prezant D.J., Nolan A. (2018). Metabolomics of World Trade Center-Lung Injury: A machine learning approach. BMJ Open Respir. Res..

[35] Engelen M.P.K.J., Rutten E.P.A., De Castro C.L.N., Wouters E.F.M., Schols A.M.W.J., Deutz N.E.P. (2007). Supplementation of soy protein with branched-chain amino acids alters protein metabolism in healthy elderly and even more in patients with chronic obstructive pulmonary disease. Am. J. Clin. Nutr..

[36] Rogers R.M., Donahoe M., Costantino J. (1992). Physiological-Effects of Oral Supplemental Feeding in Malnourished Patients with Chronic Obstructive Pulmonary-Disease—A Randomized Control Study. Am. Rev. Respir. Dis..

[37] Kutsuzawa T., Shioya S., Kurita D., Haida M. (2009). Plasma branched-chain amino acid levels and muscle energy metabolism in patients with chronic obstructive pulmonary disease. Clin. Nutr..

[38] Wang L.L., Tang Y.F., Liu S., Mao S.T., Ling Y., Liu D., He X.Y., Wang X.G. (2013). Metabonomic Profiling of Serum and Urine by H-1 NMR-Based Spectroscopy Discriminates Patients with Chronic Obstructive Pulmonary Disease and Healthy Individuals. PLoS ONE.

[39] Li J., Chen M., Lu L., Wang J., Tan L. (2022). Branched-chain amino acid transaminase 1 inhibition attenuates childhood asthma in mice by effecting airway remodeling and autophagy. Respir. Physiol. Neurobiol..

[40] Lawrence J., Nho R. (2018). The Role of the Mammalian Target of Rapamycin (mTOR) in Pulmonary Fibrosis. Int. J. Mol. Sci..

[41] Chung E.J., Sowers A., Thetford A., McKay-Corkum G., Chung S.I., Mitchell J.B., Citrin D.E. (2016). Mammalian Target of Rapamycin Inhibition with Rapamycin Mitigates Radiation-Induced Pulmonary Fibrosis in a Murine Model. Int. J. Radiat. Oncol..

[42] Scibior D., Czeczot H. (2004). Arginine--metabolism and functions in the human organism. Postepy. Hig. Med. Dosw..

[43] van Rijn J., van den Berg J., Teerlink T., Kruyt F.A., Schor D.S., Renardel de Lavalette A.C., van den Berg T.K., Jakobs C., Slotman B.J. (2003). Changes in the ornithine cycle following ionising radiation cause a cytotoxic conditioning of the culture medium of H35 hepatoma cells. Br. J. Cancer.

[44] Moroz B.B., Vasil’ev P.S., Fedorovskii L.L., Grozdov S.P., Morozova N.V. (1987). Effect of local x-ray irradiation of the abdominal area on the amino acid content of the blood plasma and their urinary excretion in dogs and rats. Radiobiologiia.

[45] Stuehr D.J. (2004). Enzymes of the L-arginine to nitric oxide pathway. J. Nutr..

[46] Ma X., Zhang Y., Jiang D., Yang Y., Wu G., Wu Z. (2019). Protective Effects of Functional Amino Acids on Apoptosis, Inflammatory Response, and Pulmonary Fibrosis in Lipopolysaccharide-Challenged Mice. J. Agric. Food Chem..

[47] Chen J., Jin Y., Yang Y., Wu Z., Wu G. (2020). Epithelial Dysfunction in Lung Diseases: Effects of Amino Acids and Potential Mechanisms. Adv. Exp. Med. Biol..

[48] Gao L., Zhang J.H., Chen X.X., Ren H.L., Feng X.L., Wang J.L., Xiao J.H. (2019). Combination of L-Arginine and L-Norvaline protects against pulmonary fibrosis progression induced by bleomycin in mice. Biomed. Pharmacother..

[49] Song L., Wang D., Cui X., Hu W. (1998). The protective action of taurine and L-arginine in radiation pulmonary fibrosis. J. Environ. Pathol. Toxicol. Oncol..

[50] Reeds P.J., Fjeld C.R., Jahoor F. (1994). Do the differences between the amino acid compositions of acute-phase and muscle proteins have a bearing on nitrogen loss in traumatic states?. J. Nutr..

[51] Speelman T., Dale L., Louw A., Verhoog N.J.D. (2022). The Association of Acute Phase Proteins in Stress and Inflammation-Induced T2D. Cells.

[52] Gao K., Mu C.L., Farzi A., Zhu W.Y. (2020). Tryptophan Metabolism: A Link Between the Gut Microbiota and Brain. Adv. Nutr..

[53] Chen Z.Y., Xiao H.W., Dong J.L., Li Y., Wang B., Fan S.J., Cui M. (2021). Gut Microbiota-Derived PGF2alpha Fights against Radiation-Induced Lung Toxicity through the MAPK/NF-kappaB Pathway. Antioxidants.

[54] Budden K.F., Gellatly S.L., Wood D.L., Cooper M.A., Morrison M., Hugenholtz P., Hansbro P.M. (2017). Emerging pathogenic links between microbiota and the gut-lung axis. Nat. Rev. Microbiol..

[55] Li Z., Shen Y., Xin J., Xu X., Ding Q., Chen W., Wang J., Lv Y., Wei X., Wei Y. (2023). Cryptotanshinone alleviates radiation-induced lung fibrosis via modulation of gut microbiota and bile acid metabolism. Phytother. Res..

[56] Hang S., Paik D., Yao L., Kim E., Trinath J., Lu J., Ha S., Nelson B.N., Kelly S.P., Wu L. (2019). Bile acid metabolites control T(H)17 and T(reg) cell differentiation. Nature.

[57] Hendrick S.M., Mroz M.S., Greene C.M., Keely S.J., Harvey B.J. (2014). Bile acids stimulate chloride secretion through CFTR and calcium-activated Cl- channels in Calu-3 airway epithelial cells. Am. J. Physiol. Lung Cell Mol. Physiol..

[58] Wu J.N., Chen J.R., Chen J.L. (2020). Role of Farnesoid X Receptor in the Pathogenesis of Respiratory Diseases. Can. Respir. J..

[59] van der Lugt B., Vos M.C.P., Grootte Bromhaar M., Ijssennagger N., Vrieling F., Meijerink J., Steegenga W.T. (2022). The effects of sulfated secondary bile acids on intestinal barrier function and immune response in an inflammatory in vitro human intestinal model. Heliyon.

[60] Benderitter M., Vincent-Genod L., Pouget J.P., Voisin P. (2003). The cell membrane as a biosensor of oxidative stress induced by radiation exposure: A multiparameter investigation. Radiat. Res..

[61] Laiakis E.C., Wang Y.W., Young E.F., Harken A.D., Xu Y., Smilenov L., Garty G.Y., Brenner D.J., Fornace A.J. (2017). Metabolic Dysregulation after Neutron Exposures Expected from an Improvised Nuclear Device. Radiat. Res..

[62] Gould R.G., Bell V.L., Lilly E.H. (1959). Stimulation of cholesterol biosynthesis from acetate in rat liver and adrenals by whole body x-irradiation. Am. J. Physiol..

[63] Feliste R., Dousset N., Carton M., Douste-Blazy L. (1981). Changes in plasma apolipoproteins following whole-body irradiation in rabbit. Radiat. Res..

[64] Beheshti A., Chakravarty K., Fogle H., Fazelinia H., Silveira W.A.D., Boyko V., Polo S.L., Saravia-Butler A.M., Hardiman G., Taylor D. (2019). Multi-omics analysis of multiple missions to space reveal a theme of lipid dysregulation in mouse liver. Sci. Rep..

[65] Gao F., Liu C., Guo J., Sun W., Xian L., Bai D., Liu H., Cheng Y., Li B., Cui J. (2015). Radiation-driven lipid accumulation and dendritic cell dysfunction in cancer. Sci. Rep..

[66] Levis G.M., Efstratiadis A.A., Mantzos J.D., Miras C.J. (1975). The effect of ionizing radiation on lipid metabolism in bone marrow cells. Radiat. Res..

[67] Calzada E., Onguka O., Claypool S.M. (2016). Phosphatidylethanolamine Metabolism in Health and Disease. Int. Rev. Cell Mol. Biol..

[68] Vazquez-de-Lara L.G., Tlatelpa-Romero B., Romero Y., Fernandez-Tamayo N., Vazquez-de-Lara F., Justo-Janeiro J.-M., Garcia-Carrasco M., de-la-Rosa Paredes R., Cisneros-Lira J.G., Mendoza-Milla C. (2018). Phosphatidylethanolamine Induces an Antifibrotic Phenotype in Normal Human Lung Fibroblasts and Ameliorates Bleomycin-Induced Lung Fibrosis in Mice. Int. J. Mol. Sci..

[69] Vishwanath V.A. (2016). Fatty Acid Beta-Oxidation Disorders: A Brief Review. Ann. Neurosci..

[70] Batchuluun B., Al Rijjal D., Prentice K.J., Eversley J.A., Burdett E., Mohan H., Bhattacharjee A., Gunderson E.P., Liu Y., Wheeler M.B. (2018). Elevated Medium-Chain Acylcarnitines Are Associated with Gestational Diabetes Mellitus and Early Progression to Type 2 Diabetes and Induce Pancreatic beta-Cell Dysfunction. Diabetes.

[71] Tarasenko T.N., Cusmano-Ozog K., McGuire P.J. (2018). Tissue acylcarnitine status in a mouse model of mitochondrial beta-oxidation deficiency during metabolic decompensation due to influenza virus infection. Mol. Genet. Metab..

[72] Goudarzi M., Weber W.M., Mak T.D., Chung J., Doyle-Eisele M., Melo D.R., Brenner D.J., Guilmette R.A., Fornace A.J. (2015). Metabolomic and lipidomic analysis of serum from mice exposed to an internal emitter, cesium-137, using a shotgun LC-MS(E) approach. J. Proteome Res..

[73] Liu H.X., Lu X., Zhao H., Li S., Gao L., Tian M., Liu Q.J. (2022). Enhancement of Acylcarnitine Levels in Small Intestine of Abdominal Irradiation Rats Might Relate to Fatty Acid beta-Oxidation Pathway Disequilibration. Dose Response.

