# Supplementary material for: Metabolomic Signatures Associated with Radiation-Induced Lung Injury by Correlating Lung Tissue to Plasma in a Rat Model
Source: Metabolites. 2023 Sep 17;13(9):1020. doi: 10.3390/metabo13091020 (PMC10536118; doi:10.3390/metabo13091020)
Supplement: Supplementary file 1 [file metabolites-13-01020-s001.zip › metabolites-2586578-supplementary.pdf]

Table S1: Concentration of internal target used for pretreatment of plasma samples.

Number	Internal standard	Concentration (μg/mL)
1	Acylcarnitine C10:0-d3	0.012
2	Acylcarnitine C16:0-d3	0.015
3	L-Citrulline-d4	8.400
4	L-Tryptophan-d5	4.000
5	L-Phenylalanine-d5	4.000
6	L-Leucine-d10	5.200
7	Succinic acid-d4	6.000
8	Cholic acid-d4 （CA-d4）	1.720
9	Chenodeoxycholic acid-d4 （CDCA-d4）	1.000
10	Stearic acid-d3	22.00
11	Palmitic acid-d3	24.00

Note: The internal standard number "1-6" is used for detection in positive ion mode of LC-MS, and the internal standard number "7-11" is used for detection in negative ion mode of LC-MS.

Table S2: Concentration of internal target used for pretreatment of lung tissues samples.

Label	Internal standard	Concentration (μg/mL)
1	Acylcarnitine C10:0-d3	0.01
2	Acylcarnitine C16:0-d3	0.01
3	L-Citrulline-d4	12.00
4	L-Tryptophan-d5	4.00
5	L-Phenylalanine-d5	4.00
6	L-Leucine-d10	5.20
7	Succinic acid-d4	6.00
8	Cholic acid-d4 （CA-d4）	1.80
9	Chenodeoxycholic acid-d4 （CDCA-d4）	2.60
10	Stearic acid-d3	24.00
11	Palmitic acid-d3	24.00

Note: The internal standard number "1-6" is used for detection in positive ion mode of LC-MS, and the internal standard number "7-11" is used for detection in negative ion mode of LC-MS.

Table S3: Statistical information of differential metabolites in lung tissues at 5d after WTI (X±SD).

Metabolite name	Control (*n*=11)	10Gy (*n*=10)	20Gy (*n*=9)	35Gy (*n*=9)
Palmitoylglycerol	5.68±1.08	6.13±1.30	6.41±1.30	7.26±0.93**, #
L-Isoleucine	1.03±0.25	0.86±0.14*	0.82±0.13**	0.79±0.11**
Cholesterol	6.41±0.69	7.81±1.02**	7.48±0.89**	7.53±0.67**
3-Amino-2-oxazolidinone	2.96±0.61	2.56±0.24*	2.41±0.35**	2.37±0.26**
4-Hydroxybenzenesulfonic acid	6.94±3.75	8.66±3.68	10.11±4.36*	4.05±1.04##, &&
Acylcarnitine C13:1	9.64±3.36	11.72±1.56*	10.63±1.39	9.30±1.32#
Acylcarnitine C15:0	1.98±0.57	2.55±0.58	3.04±0.62**	3.03±0.80**
Acylcarnitine C20:0	6.63±0.71	8.05±2.03	8.86±2.31**	9.13±1.83**
Acylcarnitine C20:1	4.12±0.59	4.65±0.94	5.33±0.81**	5.03±1.38*
Acylcarnitine C22:0	2.63±0.41	3.40±1.01	4.13±1.91**	4.33±1.05**
Acylcarnitine C5:0	5.85±1.47	4.25±0.95**	4.57±1.08*	2.97±0.90**, #, &&
Acylcarnitine C9:1	2.34±0.71	1.57±0.62**	1.36±0.58**	1.31±0.29**
Alanine	4.02±0.43	3.07±0.38**	3.60±0.80#	3.48±0.60*
Arginine	5.09±1.11	4.37±0.91	4.15±0.82*	4.19±0.49*
Ascorbic acid	0.23±0.06	0.70±0.59	0.38±0.30	0.98±0.92**, &
Asparagine	4.78±1.11	4.16±0.47	4.03±0.62*	3.93±0.44*
FFA22:5	5.08±0.83	5.18±0.89	5.63±0.63	6.15±0.73**, #
Cytosine	4.92±0.82	4.10±0.84*	4.55±1.02	3.93±0.49*
Erucic acid	6.38±1.17	5.21±0.69**	5.31±0.67**	5.49±0.32*
Indoxyl sulfate	1.89±0.44	2.88±0.73**	2.65±0.94*	1.70±0.53##, &&
Leucine	5.49±1.34	4.75±0.57	4.57±0.69*	4.47±0.50*
LPC(O-18:0)	1.58±0.26	1.91±0.26**	2.00±0.25**	1.98±0.17**
LPC(O-18:1)	1.90±0.34	2.18±0.29	2.51±0.52**	2.49±0.33**

PE(32:0)	1.73±0.41	1.43±1.23	1.11±0.30	1.06±0.40*
PE(36:5)	2.05±0.36	1.70±1.10	1.43±0.45*	1.34±0.39*
Phenylalanine	7.51±1.31	6.77±0.73	6.45±0.85*	6.35±0.69**
Proline	10.86±2.78	9.11±1.25*	9.33±1.96	8.73±1.24*
Serotonin	2.77±0.60	2.36±0.29*	2.36±0.27*	2.20±0.23**
Tauro-α-Muricholic acid	9.35±3.92	2.93±1.14**	3.52±2.23**	4.19±4.25**
Taurocholic acid	41.46±22.85	10.27±1.34**	13.83±6.43**	16.04±15.06**
Taurohyodeoxycholic Acid	8.09±3.25	2.18±0.61**	2.70±1.26**	3.02±2.27**
Threonine	5.52±1.00	4.54±0.44**	4.90±0.92	4.65±0.48*
Tryptophan	9.83±1.94	8.33±1.11*	8.31±0.96*	7.70±0.91**
Tauroursodeoxycholic acid	6.12±2.29	2.44±0.47**	2.10±0.87**	2.99±1.82**
Valine	10.92±2.51	8.81±0.90**	9.17±1.74*	8.73±1.14**
Xanthine	1.42±0.20	1.21±0.13**	1.22±0.17*	1.27±0.13
Urea	9.84±1.70	8.61±2.61	8.13±1.95	6.25±1.22**, #, &

Note: *n* represent the number of samples. LSD analysis. Compared with Control, *p<0.05, **p<0.01; Compared with 10Gy, #p<0.05, ##p<0.01; Compared with 20Gy, &p<0.05, &&p<0.01.

Table S4: Statistical information of differential metabolites in plasma at 1d after WTI (X±SD).

Metabolite name	Control (*n*=13)	10Gy (*n*=10)	20Gy (*n*=11)	35Gy (*n*=12)
Lysine	2.41±0.24	2.62±0.31	2.91±0.58**	2.81±0.31*
Glutamine	3.10±0.35	3.41±0.47	3.75±0.77**	3.69±0.44*
Tryptophan	2.52±0.26	2.35±0.27	2.25±0.42*	2.64±0.28$^{\#, \&\&}$
Tyrosine	3.58±0.67	4.37±0.71*	4.56±0.78**	4.74±0.77**
Glycine	6.47±3.83	3.33±2.60*	6.26±3.20$^{\#}$	2.45±0.86$^{**, \#\#}$
Cytosine	0.95±0.13	1.38±0.14**	1.61±0.21$^{**, \#\#}$	1.54±0.13$^{**, \#}$
Adenine	3.35±0.10	3.42±0.14	3.42±0.11	3.31±0.08$^{\#, \&}$
Ascorbic acid	2.01±0.35	2.01±0.36	1.94±0.44	2.78±0.78$^{**, \#\#, \&\&}$
Acylcarnitine C12:1	3.59±1.06	5.19±1.31	6.75±3.89**	5.30±0.80
Acylcarnitine C13:1	4.24±1.49	5.68±1.36	6.26±2.98*	4.68±0.89$^{\&}$
Acylcarnitine C14:0	5.11±1.48	6.81±2.82	8.13±3.99*	7.99±1.75*
Acylcarnitine C14:1	4.08±1.19	5.20±1.82	7.27±4.68**	5.86±1.16
Acylcarnitine C15:0	0.75±0.30	1.02±0.47	1.32±0.48**	1.23±0.28**
Acylcarnitine C16:0	0.57±0.21	0.80±0.26	0.93±0.49*	1.04±0.24**
Acylcarnitine C16:3	1.07±0.31	1.42±0.52	2.15±1.62**	1.72±0.30
Acylcarnitine C18:0	2.62±1.14	3.32±1.68	4.38±1.95**	4.70±0.77$^{**, \#}$
Acylcarnitine C18:2	0.64±0.20	0.81±0.33	0.99±0.40**	0.97±0.18*
Acylcarnitine C20:0	1.57±0.63	1.80±1.13	2.92±0.95$^{**, \#\#}$	2.77±0.74$^{**, \#}$
Acylcarnitine C20:1	1.94±0.82	2.60±0.70	2.95±0.72**	3.74±0.76$^{**, \#\#, \&}$
Acylcarnitine C20:4	1.31±0.34	1.65±0.48	2.50±1.58$^{**, \#}$	2.23±0.31*
Acylcarnitine C6:0	7.94±1.89	10.03±3.41	10.95±3.61*	11.27±2.95*
Acylcarnitine C8:0	6.19±1.57	7.60±1.93	8.42±2.63*	9.75±2.68$^{**, \#}$
FFA22:5	1.32±0.31	1.68±0.30**	1.37±0.24$^{\#\#}$	1.44±0.22$^{\#}$

FFA21:0	1.48±0.51	1.50±0.34	1.02±0.32**, ##	1.24±0.38
Indole-3-Lactic acid	0.87±0.22	1.09±0.43	1.81±0.95**, #	2.53±0.87**, ##, &
Indole	5.61±0.45	5.22±0.48	4.95±0.88**	5.70±0.59&&
PC(31:0)	2.56±0.82	2.84±1.12	3.65±0.98**	3.06±0.85
LPE(16:0)	1.33±0.24	1.50±0.30	1.75±0.22**, #	1.95±0.30**, ##
LPE(17:0)	5.42±1.41	5.79±1.95	7.21±1.17*, #	7.20±1.86*, #
LPE(18:0)	2.88±0.71	2.82±0.90	3.67±0.62*, #	3.86±0.88**, ##
Pyruvate	1.58±0.38	1.99±0.55	2.15±0.41*	2.61±0.64**, &
Serotonin	2.99±0.29	2.77±0.31	2.59±0.49*	3.05±0.29&&
Spermine	0.90±0.21	0.86±0.21	0.97±0.31	1.17±0.33*, #
2,3-Dihydroxybenzoate	4.64±1.81	3.30±1.97	2.46±1.86**	1.50±0.57**, #
3-Methylquinoline	1.02±0.09	0.94±0.09	0.90±0.17*	1.04±0.10&&
DL-P-Hydroxyphenyl lactic acid	2.11±0.35	2.69±0.43	3.63±2.03**	3.54±0.46**
Benzophenone	2.06±0.85	1.05±0.42**	1.54±0.66	1.72±0.63#
β-Nicotyrine	5.22±0.53	4.81±0.43	4.59±0.85*	5.26±0.55&
Perfluorooctanesulfonic acid	7.57±1.06	8.55±0.97	7.58±0.75	8.99±1.79**, &&
Urea	5.62±1.44	4.97±0.94	4.07±0.63*, #	3.16±0.77**, ##, &

Note: *n* represent the number of samples. LSD analysis. Compared with Control, *p<0.05, **p<0.01; Compared with 10Gy, #p<0.05, ##p<0.01; Compared with 20Gy, &p<0.05, &&p<0.01.

Table S5: Statistical information of differential metabolites in plasma at 2d after WTI (X±SD).

Metabolite name	Control (*n*=13)	10Gy (*n*=10)	20Gy (*n*=11)	35Gy (*n*=12)
Phenylalanine	3.07±0.28	2.81±0.28*	2.71±0.21**	2.88±0.33
Pro-Leu	4.68±0.68	3.90±0.66*	3.88±0.57*	4.52±1.03
Proline	1.61±0.36	1.25±0.21**	1.34±0.23*	1.25±0.22**
Kynurenic acid	4.69±0.57	4.22±0.39*	4.14±0.64*	3.99±0.51**
Lysine	2.33±0.47	2.73±0.54	3.01±0.50**	2.81±0.45*
Methionine	1.01±0.11	0.76±0.10**	0.91±0.16$^{\#}$	0.90±0.18$^{\#}$
Glutamine	2.92±0.51	3.39±0.69	3.68±0.70**	3.60±0.54**
Glycine	7.56±2.23	2.44±1.00	9.56±12.09$^{\#}$	3.34±2.38$^{\&}$
Hippuric acid	5.84±1.45	3.47±1.22**	2.14±0.62$^{**, \#}$	3.55±1.42$^{**, \&\&}$
Histidine	0.98±0.14	1.05±0.10	1.00±0.10	0.89±0.14$^{\#\#, \&}$
Threonine	3.22±0.50	2.70±0.53	3.80±1.09$^{*, \#\#}$	2.79±0.55$^{\&\&}$
Tryptophan	2.27±0.29	2.06±0.21	1.98±0.28*	2.05±0.26*
Tyrosine	3.85±0.88	4.38±0.52	4.91±0.44**	4.92±1.34**
Cytosine	8.46±1.01	7.10±0.72**	6.91±0.84**	6.21±0.69$^{**, \#}$
Acylcarnitine C10:0	3.97±1.00	6.87±1.19**	6.98±1.55**	7.05±1.58**
Acylcarnitine C10:1	1.61±0.40	2.43±0.43**	2.37±0.46**	2.35±0.44**
Acylcarnitine C10:2	1.17±0.24	1.51±0.20**	1.46±0.28**	1.40±0.19*
Acylcarnitine C12:0	10.74±2.54	16.17±2.34**	15.93±2.42**	16.96±3.64**
Acylcarnitine C12:1	3.11±0.80	4.39±0.67**	4.73±0.77**	4.57±1.14**
Acylcarnitine C14:0	4.28±1.24	6.45±0.79**	6.76±0.88**	7.62±2.19**
Acylcarnitine C14:2	4.69±1.59	7.52±1.09**	7.25±0.99**	7.74±1.86**
Acylcarnitine C15:0	0.65±0.21	1.06±0.23**	1.20±0.17**	1.20±0.32**
Acylcarnitine C16:0	4.06±0.95	6.42±0.76**	6.96±1.16**	7.94±2.05$^{**, \#}$

Acylcarnitine C16:1	1.42±0.51	2.43±0.35**	2.50±0.40**	2.84±0.82**
Acylcarnitine C16:2	1.57±0.60	2.62±0.46**	2.62±0.44**	3.09±1.01**
Acylcarnitine C16:3	0.90±0.33	1.49±0.28**	1.44±0.23**	1.49±0.31**
Acylcarnitine C16:4	1.23±0.35	1.81±0.31**	1.65±0.29**	1.73±0.24**
Acylcarnitine C18:0	2.70±0.47	4.15±0.53**	5.23±1.03**, #	5.41±1.56**, ##
Acylcarnitine C18:1	3.73±1.49	5.91±0.82**	6.42±1.21**	7.56±1.92**, #
Acylcarnitine C18:2	4.14±1.50	6.68±1.07**	7.10±0.95**	8.07±2.36**, #
Acylcarnitine C18:3	3.17±1.13	5.27±1.07**	5.32±0.83**	6.07±1.42**
Acylcarnitine C20:0	1.16±0.39	1.73±0.18**	2.51±0.55**, ##	2.59±0.62**, ##
Acylcarnitine C20:1	1.17±0.37	1.79±0.29**	2.36±0.53**, ##	2.35±0.44**, ##
Acylcarnitine C20:4	1.05±0.36	1.66±0.23**	1.70±0.23**	1.76±0.33**
Acylcarnitine C5:0	10.28±1.27	7.12±0.84**	7.84±3.44**	5.71±1.64**, &
Acylcarnitine C6:0	4.53±1.41	8.16±1.61**	8.75±2.35**	8.18±1.87**
Acylcarnitine C2:0	2.00±0.21	2.27±0.21*	2.51±0.51**	2.35±0.26**
Acylcarnitine C4:0	2.17±0.41	1.92±0.26	1.80±0.56*	1.71±0.31**
Acylcarnitine C8:0	3.81±1.15	6.60±1.34**	7.10±1.72**	6.87±1.63**
β-Linoleic acid	2.95±1.32	4.14±1.18	5.15±1.91**	4.68±1.70**
Linolenic acid	2.93±1.31	4.08±1.40	4.72±1.75**	4.44±1.73*
Erucic acid	6.46±1.89	7.91±1.89	7.99±2.17	6.08±1.79*, &
Palmitic acid	1.57±0.24	2.07±0.35*	2.38±0.59**	2.07±0.54**
Oleic acid	1.55±0.50	2.35±0.63*	3.03±1.19**	2.67±1.01**
FFA20:2	0.77±0.23	1.07±0.25	1.38±0.59**	1.20±0.43*
FFA20:3	0.73±0.23	1.13±0.30*	1.33±0.54**	1.18±0.47**
FFA22:5	2.70±0.88	4.05±1.02*	4.88±2.19**	4.63±1.89**
FFA22:6	0.63±0.22	0.82±0.18	0.98±0.41**	0.94±0.36*

Glycohyodeoxycholic acid	1.14±1.36	1.07±1.04	0.94±0.71	2.16±1.07*, #, &
Deoxycholic acid	2.33±1.76	1.86±0.76	3.12±1.36	5.63±3.59**, ##, &&
Indole-3-Lactic acid	7.26±1.28	5.14±1.18**	5.27±1.77**	4.64±0.73**
Indole	5.35±0.70	4.94±0.40	4.69±0.54**	4.86±0.59*
LPA(14:0)	3.92±2.41	2.75±0.40*	2.50±0.50*	2.39±0.52**
LPC(O-18:1)	2.82±0.44	2.51±0.24	2.90±0.41#	2.59±0.31&
LPC(18:2)	2.45±0.14	2.13±0.10**	2.31±0.38	2.19±0.10**
LPC(20:3)	2.53±0.40	2.34±0.21	2.41±0.46	2.10±0.37**
LPE(16:0)	1.71±0.14	1.87±0.16	2.11±0.30**, #	2.24±0.33**, ##
LPE(17:0)	7.21±1.90	7.54±1.04	9.11±1.35**, #	8.29±1.36
LPE(18:0)	3.54±0.57	3.67±0.47	4.36±0.81**, #	4.31±0.69**, #
PC(O-36:5)	1.28±0.27	1.22±0.08	1.90±0.78**, ##	1.23±0.09&&
PC(O-36:6)	5.09±1.37	4.01±0.58**	4.46±0.90	4.20±0.61*
PC(O-38:4)	7.02±1.78	9.90±0.99**	9.17±0.82**	8.26±1.13*, ##
PC(34:0)	0.88±0.22	0.91±0.07	1.10±0.17**, #	1.03±0.12*
PC(34:3)	2.31±0.56	2.55±0.30	3.17±1.02**, #	2.67±0.46
PC(38:1)	2.15±0.46	2.35±0.25	2.87±0.48**, ##	2.38±0.20&&
PC(38:5)	4.03±0.72	5.15±0.35**	4.67±0.30**, #	4.57±0.62*, #
PC(38:7)	1.90±0.42	2.49±0.23**	2.34±0.32*	2.45±0.53**
PC(39:4)	0.93±0.24	1.29±0.17**	1.23±0.17**	1.19±0.14**
PC(40:6)	5.48±0.98	6.59±0.79**	5.91±0.47#	6.08±0.63
PE(36:3)	2.28±0.78	1.59±0.33*	2.62±1.30##	2.00±0.46
PE(36:4)	5.17±1.60	4.97±0.99	6.68±2.12*, #	6.52±1.28*, #
PE(36:5)	1.19±0.24	0.95±0.09**	1.14±0.25#	1.14±0.15#
PE(38:4)	3.13±0.93	4.28±1.02*	5.50±1.78**, #	5.37±1.33**

Serotonin	2.50±0.34	2.35±0.18	2.19±0.24**	2.28±0.27*
Spermine	7.27±1.23	7.40±1.18	8.02±1.26	9.15±2.53**, #
1-Aminocyclopropanecarboxylic acid	5.47±0.92	4.68±0.61*	4.08±1.07**	4.68±0.97*
2,3-Dihydroxybenzoate	5.47±1.88	2.06±0.72**	1.01±0.46**, #	1.42±0.86**
3-Amino-2- oxazolidone	2.48±0.25	2.35±0.17	2.19±0.20**	2.36±0.29
3-Methylquinoline	1.02±0.13	0.95±0.07	0.89±0.11**	0.93±0.12*
Benzophenone	2.41±1.45	0.99±0.47**	0.63±0.38**	1.28±0.51**
3-Aminobutyric acid	6.04±0.80	6.20±0.77	5.88±0.97	5.34±0.96#
DL-P-Hydroxyphenyl lactic acid	1.74±0.34	2.02±0.34	1.93±0.46	2.19±0.33**
D-Pipecolinic acid	2.86±0.33	3.24±0.38*	3.30±0.34**	3.28±0.33**
Urea	6.89±0.91	5.02±0.77**	5.85±2.17	4.43±1.66**

Note: *n* represent the number of samples. LSD analysis. Compared with Control, *p<0.05, **p<0.01; Compared with 10Gy, #p<0.05, ##p<0.01; Compared with 20Gy, &p<0.05, &&p<0.01.

Table S6: Statistical information of differential metabolites in plasma at 3d after WTI (X±SD).

Metabolite name	Control (*n*=13)	10Gy (*n*=10)	20Gy (*n*=11)	35Gy (*n*=12)
Tryptophan	2.60±0.31	2.40±0.17	2.13±0.29**, #	1.98±0.31**, ##
Tyrosine	3.76±0.50	4.10±0.79	5.08±1.05**, ##	4.64±0.53**
Valine	3.65±0.62	3.49±0.40	3.23±0.63	2.84±0.37**, ##
Proline	2.31±0.45	2.17±0.26	1.80±0.20**, ##	1.75±0.12**, ##
Serine	4.01±0.52	4.26±1.20	3.67±0.72	3.35±0.66*, ##
Phenylalanine	3.61±0.30	3.63±0.58	3.23±0.30*, #	2.80±0.33**, ##, &
Ornithine	1.19±0.20	1.05±0.12	0.98±0.16**	0.86±0.16**, ##
Methionine	1.29±0.23	1.18±0.17	0.97±0.17**, #	0.93±0.09**, ##
Kynurenic acid	5.07±0.63	5.19±0.70	4.47±0.44*, ##	3.91±0.41**, ##, &
Leucine	7.68±1.28	6.81±0.83	6.65±1.07*	5.68±1.00**, #, &
Glycine	9.70±5.34	11.89±5.77	4.94±2.34**, ##	2.82±1.25**, ##
Asparagine	3.97±0.68	3.60±0.42	3.47±0.48*	3.04±0.53**, #
Cytosine	1.59±0.17	1.50±0.21	1.24±0.18**, ##	1.29±0.13**, ##
Aspartic acid	2.52±0.33	2.37±0.30	2.26±0.33	2.10±0.31**
Hippuric acid	5.10±1.83	5.40±2.75	4.07±1.12	3.11±0.83**, ##
Histidine	1.20±0.09	1.26±0.06	1.18±0.16	0.98±0.13**, ##, &&
Hydroxyproline	1.62±0.21	1.46±0.14	1.40±0.21**	1.18±0.18**, ##, &&
Adenine	3.61±0.13	3.56±0.11	3.52±0.12	3.47±0.11**
Acylcarnitine C10:0	5.69±1.97	7.42±2.35*	7.69±1.16*	8.25±2.17**
Acylcarnitine C10:1	2.23±0.61	2.79±0.42**	2.89±0.51**	2.63±0.36*
Acylcarnitine C10:2	1.52±0.30	1.83±0.19**	1.65±0.27	1.48±0.21##
Acylcarnitine C12:1	3.94±1.36	5.15±1.76*	5.30±1.04*	5.08±1.17*
Acylcarnitine C14:2	0.64±0.27	0.87±0.45	0.92±0.19*	0.92±0.17*

Acylcarnitine C15:0	0.81±0.39	1.07±0.64	1.19±0.31*	1.41±0.34**
Acylcarnitine C16:0	4.82±1.41	5.82±3.49	6.05±1.17	8.24±1.54**, ##, &
Acylcarnitine C16:1	2.07±0.92	2.72±1.95	3.05±0.69*	3.36±0.78**
Acylcarnitine C18:0	2.24±0.37	3.00±1.23	3.39±1.16**	4.09±0.88**, ##
Acylcarnitine C18:1	0.53±0.26	0.79±0.50	0.75±0.21	1.12±0.27**, #, &&
Acylcarnitine C18:2	0.66±0.32	0.83±0.51	1.00±0.27*	1.08±0.20**
Acylcarnitine C18:3	4.20±2.17	5.56±3.37	6.64±2.08*	6.56±1.42*
Acylcarnitine C20:0	0.67±0.16	0.96±0.32	1.25±0.59**	1.68±0.71**, ##, &
Acylcarnitine C20:1	1.48±0.61	2.01±0.78	2.31±0.74**	3.20±0.64**, ##, &&
Acylcarnitine C20:4	1.53±0.55	1.99±0.83	2.40±0.54**	2.39±0.39**
Acylcarnitine C5:0	13.74±3.31	14.59±2.67	9.95±2.47**, ##	6.65±1.68**, ##, &&
Acylcarnitine C6:0	0.71±0.26	0.95±0.30*	0.97±0.19*	0.95±0.22*
Acylcarnitine C9:1	5.49±1.13	6.18±1.05	4.90±1.22##	4.02±0.73**, ##, &
Acylcarnitine C2:0	2.72±0.38	3.07±0.42*	3.11±0.28**	2.95±0.23
Acylcarnitine C3:0	1.02±0.16	1.11±0.25	0.95±0.19#	0.77±0.09**, ##, &
Acylcarnitine C4:0	2.37±0.43	2.38±0.45	2.13±0.58	1.54±0.25**, ##, &&
Acylcarnitine C6:1	1.50±0.21	1.70±0.27	1.42±0.30#	1.34±0.19##
Acylcarnitine C8:0	55.50±17.12	75.08±21.50*	77.62±13.51**	79.45±20.01**
Acylcarnitine C8:1	3.63±0.82	3.68±0.58	2.94±0.77*, #	2.17±0.52**, ##, &
Choline	2.15±0.36	2.16±0.27	1.82±0.36*, #	1.66±0.23**, ##
N-Acetylneuraminic acid	1.49±0.73	1.43±0.49	0.84±0.46**, #	1.01±0.36*
Erucamide	7.12±3.85	5.30±1.93	10.15±9.12#	3.28±0.73&&
Erucic acid	5.13±1.03	5.97±0.91	8.09±2.65**, ##	5.99±1.20&&
Palmitic acid	1.85±0.50	1.91±0.54	2.65±0.65**, ##	2.24±0.19
Myristic acid	0.70±0.24	0.74±0.24	1.03±0.33**, #	0.81±0.14&

Biliverdin	1.98±0.66	1.88±0.55	1.20±0.46[**, ##]	1.99±0.47[&&]
β-Linoleic acid	3.80±1.54	3.66±1.79	6.17±1.99[**, ##]	5.23±1.21[*, #]
Linolenic acid	3.72±1.49	3.61±1.70	5.85±2.07[**, ##]	4.91±1.26
FFA20:0	1.64±0.81	1.47±0.55	1.85±0.60	3.20±1.57[**, ##, &&]
Oleic acid	1.95±0.77	1.88±0.97	3.24±1.06[**, ##]	2.59±0.64
FFA19:0	0.64±0.29	0.57±0.18	0.74±0.19	1.11±0.49[**, ##, &&]
FFA20:2	0.89±0.31	0.81±0.34	1.43±0.46[**, ##]	1.11±0.30[&]
FFA20:3	1.08±0.37	0.96±0.41	1.70±0.55[**, ##]	1.34±0.42[#]
FFA20:4	2.67±0.49	2.46±0.56	3.10±0.51[##]	2.93±0.55[#]
FFA22:5	3.17±1.10	3.06±1.39	4.79±1.20[**, ##]	4.50±0.90[**, ##]
FFA22:6	0.76±0.25	0.74±0.29	1.07±0.23[**, ##]	0.95±0.19[#]
FFA21:0	4.89±2.86	4.12±1.60	5.67±2.16	9.11±4.80[**, ##, &]
Glycohyodeoxycholic acid	1.34±1.85	2.97±2.36[*]	2.90±1.62[*]	3.19±1.24[*]
Deoxycholic acid	3.40±1.82	4.78±2.36	4.79±2.49	6.95±3.75[**]
Tauro-α-Muricholic acid	5.28±1.87	3.36±1.81[**]	2.42±1.39[**]	1.92±1.56[**]
Taurohyodeoxycholic acid	6.45±2.04	4.63±2.48	2.82±2.04[**]	4.54±4.13
Pantothenic acid	2.22±0.37	2.29±0.36	1.98±0.24	1.98±0.42[#]
Indole-3-Lactic acid	6.61±1.27	5.85±1.85	4.98±1.03[**]	5.13±1.65[*]
Indole	5.25±0.58	5.03±0.50	4.35±0.47[**, ##]	3.93±0.57[**, ##]
Indoxyl sulfate	1.77±0.34	2.51±0.65[**]	1.58±0.45[##]	1.37±0.39[*, ##]
LPA(14:0)	4.46±0.86	3.64±1.18[*]	3.41±0.54[**]	2.92±0.76[**]
LPA(20:1)	2.78±0.54	2.16±0.59[**]	3.07±0.46[##]	2.44±0.44[&&]
LPC(O-18:0)	3.64±0.46	3.65±0.71	4.37±0.72[**, #]	3.92±0.63
LPC(O-18:1)	2.81±0.29	2.33±0.49[**]	2.90±0.33[##]	2.37±0.22[**, &&]
LPC(16:0)	1.45±0.14	1.30±0.20[*]	1.62±0.13[*, ##]	1.46±0.21[#, &]

LPC(18:2)	2.46±0.15	2.17±0.31**	2.30±0.21	2.11±0.16$^{**, \&}$
LPC(20:3)	2.73±0.37	2.12±0.56**	2.60±0.56$^{\#}$	1.98±0.40$^{**, \&\&}$
LPE(16:0)	0.93±0.11	1.00±0.18	1.07±0.27	1.18±0.19$^{**, \#}$
LPE(20:2)	1.66±0.13	1.36±0.23**	1.33±0.16**	1.29±0.25**
PC(O-36:5)	1.09±0.13	1.15±0.20	1.16±0.13	0.96±0.16$^{\#\#, \&\&}$
PC(O-36:6)	4.61±0.94	3.94±0.95	4.43±0.80	3.04±0.61$^{**, \#, \&\&}$
PC(O-38:4)	0.65±0.13	0.75±0.11	0.82±0.15**	0.69±0.18$^{\&}$
PC(34:0)	7.10±1.74	6.46±1.06	8.90±2.31$^{*, \#\#}$	7.03±1.90$^{\&}$
PC(34:3)	2.43±0.54	2.27±0.36	2.87±0.72$^{\#}$	2.22±0.48$^{\&\&}$
PC(35:1)	3.80±0.71	3.58±0.56	4.39±0.87$^{\#}$	3.31±0.85$^{\&\&}$
PC(36:3)	3.01±0.37	2.66±0.36*	2.97±0.40	2.34±0.35$^{**, \#, \&\&}$
PC(36:5)	4.08±0.82	3.66±0.64	4.16±1.22	3.13±0.64$^{**, \&\&}$
PC(38:1)	2.09±0.22	2.30±0.40	2.59±0.29$^{**, \#}$	2.10±0.28$^{\&\&}$
PC(38:5)	4.60±0.63	4.64±0.41	5.24±0.80$^{*, \#}$	4.45±0.64$^{\&\&}$
PC(38:7)	2.15±0.39	2.34±0.42	3.08±0.68$^{**, \#\#}$	2.46±0.48$^{\&\&}$
PC(39:4)	7.76±1.89	8.72±1.77	10.47±2.34**	8.47±2.83$^{\&}$
PC(40:6)	6.27±0.83	6.36±0.91	7.21±0.86$^{*, \#}$	6.08±0.86$^{\&\&}$
PC(40:8)	2.55±0.35	2.37±0.50	3.07±0.59$^{*, \#\#}$	2.33±0.45$^{\&\&}$
PE(36:3)	1.68±0.48	1.21±0.34**	1.45±0.40	1.11±0.31$^{**, \&}$
PE(36:5)	1.19±0.18	1.00±0.14**	1.07±0.16	0.93±0.14$^{**, \&}$
PE(38:4)	2.38±0.65	2.02±0.55	3.08±1.23$^{*, \#\#}$	2.77±0.80$^{\&}$
Piperidine	4.15±0.41	3.97±0.40	3.58±0.50**	2.81±0.48$^{**, \#\#, \&\&}$
Pyruvate	1.95±0.32	2.08±0.30	1.90±0.31	2.41±0.45$^{**, \#, \&\&}$
Serotonin	2.92±0.34	2.81±0.27	2.38±0.21$^{**, \#\#}$	2.24±0.28$^{**, \#\#}$
Spermine	0.70±0.08	0.76±0.14	0.75±0.11	1.00±0.37$^{**, \#, \&\&}$

DL-P-Hydroxyphenyl lactic acid	1.33±0.23	1.52±0.23	1.69±0.32**	1.74±0.33**
2,3-Dihydroxybenzoate	4.18±0.96	4.48±3.11	2.64±1.09*, #	1.19±0.53**, ##, &
2-Hydroxybutyric acid	3.88±0.67	4.17±1.27	3.40±0.55#	3.08±0.50*, ##
3-Amino-2- oxazolidone	2.29±0.21	2.39±0.38	2.10±0.22#	1.74±0.21**, ##, &&
3-Aminobutyric acid	7.03±1.09	7.21±0.91	5.82±0.97**, ##	5.46±0.83**, ##
3-Methylquinoline	9.88±1.10	9.48±0.95	8.28±0.87**, ##	7.41±1.01**, ##, &
4-Hydroxybenzenesulfonic acid	6.48±1.76	11.53±6.53**	6.34±3.31##	4.62±1.94##
4-Pyridoxic acid	7.72±2.36	10.64±3.76*	6.59±2.49##	5.94±1.88##
5-Hydroxyindole	1.74±0.67	3.35±1.42**	1.30±0.66##	1.11±0.40##
β-Nicotyrine	4.98±0.55	4.72±0.48	4.09±0.45**, ##	3.71±0.51**, ##
Urea	5.95±0.34	4.93±1.08*	4.50±1.36*	3.16±0.79**, ##, &

Note: *n* represent the number of samples. LSD analysis. Compared with Control, *p<0.05, **p<0.01; Compared with 10Gy, #p<0.05, ##p<0.01; Compared with 20Gy, &p<0.05, &&p<0.01.

Table S7: Statistical information of differential metabolites in plasma at 5d after WTI (X±SD).

Metabolite name	Control (*n*=13)	10Gy (*n*=10)	20Gy (*n*=11)	35Gy (*n*=12)
Valine	3.67±0.54	3.49±0.44	3.40±0.46	2.73±0.51$^{**, \#\#, \&\&}$
Betaine	6.34±0.93	7.35±0.61**	7.81±0.64**	7.24±0.97**
Arginine	2.55±0.41	2.31±0.35	2.21±0.41*	2.47±0.29
Asparagine	4.51±0.58	3.88±0.41*	3.46±0.41**	2.90±0.78$^{**, \#\#, \&}$
Tryptophan	2.77±0.37	2.37±0.27**	2.31±0.29**	1.91±0.27$^{**, \#\#, \&\&}$
Leucine	8.36±0.97	7.30±0.84*	6.56±0.56**	5.50±1.34$^{**, \#\#, \&}$
Tyrosine	3.61±0.53	4.34±0.74**	4.34±0.42**	4.29±0.47**
Methionine	1.20±0.14	1.13±0.20	0.97±0.10$^{**, \#\#}$	0.98±0.11$^{**, \#}$
Ornithine	1.22±0.22	1.12±0.16	1.07±0.18	0.90±0.17$^{**, \#\#, \&}$
Serine	4.22±0.70	3.92±0.71	3.93±0.58	3.25±0.66$^{**, \#, \&}$
Citrulline	0.94±0.10	1.04±0.14*	1.07±0.12**	0.99±0.11
Kynurenic acid	5.12±0.48	4.45±0.29**	4.02±0.45**	3.61±0.68$^{**, \#\#}$
Glycine	12.95±5.45	8.49±5.79*	8.69±3.50*	4.65±3.45$^{**, \&}$
Histidine	1.05±0.07	1.10±0.11	0.98±0.07$^{\#\#}$	0.86±0.15$^{**, \#\#, \&}$
Phenylalanine	3.55±0.36	3.22±0.22*	3.03±0.31**	2.60±0.43$^{**, \#\#, \&\&}$
Hydroxyproline	1.69±0.22	1.46±0.14*	1.49±0.41	1.17±0.20$^{**, \#, \&\&}$
Cytosine	1.76±0.17	1.72±0.19	1.49±0.16$^{**, \#\#}$	1.31±0.11$^{**, \#\#, \&}$
Pro-Leu	4.50±0.56	4.92±0.43	4.81±0.69	5.44±0.79$^{**, \&}$
Uridine	4.07±0.87	4.82±0.77*	4.76±0.58*	4.95±0.93**
Acylcarnitine C10:0	0.53±0.16	0.65±0.16	0.64±0.09	0.81±0.21$^{**, \#, \&}$
Acylcarnitine C10:1	2.17±0.61	2.72±0.51*	2.66±0.23*	2.99±0.65**
Acylcarnitine C12:0	1.30±0.35	1.57±0.37	1.53±0.22	1.77±0.40**
Acylcarnitine C12:1	4.00±1.13	5.38±1.03*	5.91±1.31**	5.50±1.48**

Acylcarnitine C13:1	5.29±1.63	6.81±1.23**	7.17±1.09**	5.41±0.82#, &&
Acylcarnitine C14:0	0.57±0.16	0.67±0.16	0.69±0.13	0.80±0.21**
Acylcarnitine C14:1	4.37±1.48	5.68±1.33*	6.14±1.00**	6.67±1.73**
Acylcarnitine C14:2	0.58±0.19	0.79±0.17**	0.84±0.11**	0.92±0.22**
Acylcarnitine C15:0	0.78±0.26	1.15±0.31*	1.31±0.44**	1.28±0.28**
Acylcarnitine C16:0	4.74±1.15	5.79±1.21	6.14±1.12*	7.38±1.66**, ##, &
Acylcarnitine C16:1	1.96±0.73	2.37±0.66	2.57±0.48*	2.88±0.81**
Acylcarnitine C16:2	2.02±0.76	2.72±0.63*	2.97±0.48**	3.28±0.89**
Acylcarnitine C16:3	1.05±0.37	1.47±0.34**	1.54±0.22**	1.60±0.32**
Acylcarnitine C16:4	1.47±0.45	1.81±0.42*	1.97±0.29**	1.83±0.28*
Acylcarnitine C18:0	2.10±0.53	2.58±0.43	2.93±1.13*	3.51±0.97**, #
Acylcarnitine C18:1	0.49±0.17	0.64±0.11*	0.72±0.12**	0.81±0.18**, #
Acylcarnitine C18:2	0.75±0.27	1.00±0.21*	1.16±0.19**	1.21±0.26**, #
Acylcarnitine C18:3	3.82±1.36	4.97±1.14*	5.52±0.86**	5.76±1.63**
Acylcarnitine C20:0	1.36±0.45	2.08±0.52*	2.31±1.17**	2.83±1.00**, #
Acylcarnitine C20:1	1.92±0.64	2.80±0.45**	3.20±0.47**	3.44±0.65**, #
Acylcarnitine C20:4	1.53±0.48	1.97±0.39**	2.17±0.25**	2.19±0.37**
Acylcarnitine C5:0	11.81±1.68	10.99±2.60	9.03±1.92**, #	7.07±1.81**, ##, &
Acylcarnitine C6:0	0.63±0.22	0.76±0.20	0.79±0.14	0.93±0.26**
Acylcarnitine C9:1	6.82±1.41	6.20±1.48	5.45±1.44*	4.92±0.89**, #
Acylcarnitine C2:0	2.43±0.31	2.63±0.25	2.61±0.18	2.75±0.23**
Acylcarnitine C4:0	2.09±0.21	1.98±0.44	1.66±0.45**, #	1.54±0.19**, ##
Acylcarnitine C8:0	5.09±1.58	6.31±1.55	6.09±0.86	7.81±2.12**, #, &
Acylcarnitine C8:1	3.84±0.74	3.54±0.82	3.08±0.88*	2.44±0.52**, ##, &
Gluconic acid	1.06±0.41	1.63±0.54*	1.58±0.72*	1.84±0.69**

Citric Acid	4.53±0.67	5.15±1.06	3.92±0.55$^{\#\#}$	4.75±0.94$^{\&}$
Malic acid	1.94±0.48	3.43±1.78**	2.16±1.29$^{\#}$	2.07±0.82$^{\#\#}$
Lactic acid	5.15±1.10	7.21±2.44**	5.49±1.27$^{\#}$	6.20±1.77
α-Ketoglutaric acid	4.98±0.92	5.50±0.68	4.91±0.98	4.55±0.63$^{\#\#}$
Succinic acid	0.70±0.13	1.57±1.40*	0.96±1.00	0.83±0.44$^{\#}$
Creatinine	1.28±0.12	1.38±0.15*	1.22±0.11$^{\#\#}$	1.37±0.11$^{\&\&}$
Choline	2.21±0.26	2.19±0.33	1.96±0.24*	1.56±0.26$^{**, \#\#, \&\&}$
Acetylcholine	9.42±1.35	7.76±1.40**	7.21±1.08**	6.82±1.35**
FFA22:5	1.11±0.24	1.26±0.22	1.47±0.47*	1.40±0.34*
Oleic acid	1.80±0.57	2.07±0.58	2.13±0.43	2.72±0.95$^{**, \#, \&}$
β-Linoleic acid	3.37±1.13	4.06±1.01	4.11±0.77	5.33±1.90$^{**, \#, \&}$
FFA20:2	0.87±0.24	1.05±0.31	1.06±0.17	1.23±0.42**
FFA20:3	0.99±0.31	1.12±0.31	1.12±0.24	1.40±0.51**
Linolenic acid	3.28±1.09	3.86±1.14	3.93±0.97	4.83±1.67**
LPA(14:0)	4.94±0.63	4.13±0.97*	3.50±1.11**	2.90±0.38$^{**, \#\#}$
LPA(20:1)	2.66±0.44	2.25±0.35**	1.98±0.31**	2.17±0.31**
LPC(O-18:1)	3.08±0.34	2.76±0.36*	2.51±0.28**	2.51±0.28**
LPC(18:2)	2.61±0.27	2.44±0.18	2.40±0.24*	2.33±0.14**
LPC(20:3)	2.85±0.46	2.22±0.39**	2.03±0.42**	1.93±0.29**
PC(O-36:5)	1.16±0.16	1.41±0.09**	1.23±0.15$^{\#\#}$	1.17±0.12$^{\#\#}$
PC(O-36:6)	5.21±0.71	5.45±0.62	4.72±0.94$^{\#}$	4.23±0.70$^{**, \#\#}$
PC(O-38:4)	0.72±0.27	1.08±0.14**	0.98±0.14**	0.98±0.21**
PC(34:3)	2.61±0.38	3.27±0.69*	3.20±0.52	3.40±1.16*
PC(38:1)	2.15±0.39	2.87±0.28**	2.56±0.37$^{**, \#}$	2.63±0.34**
PC(38:7)	2.15±0.28	2.91±0.35**	3.12±0.49**	3.42±1.15**

PC(39:4)	0.92±0.40	1.44±0.17[**]	1.19±0.21[*]	1.32±0.41[**]
PE(38:4)	2.40±0.58	2.49±0.57	2.80±0.98	3.97±2.09[**, ##, &]
PI(18:1/22:6)	1.12±0.31	2.06±0.42[**]	1.60±0.36[**, #]	1.74±0.46[**]
PI(36:4)	2.54±0.40	3.41±0.38[**]	2.65±0.61[##]	2.91±0.74[#]
PI(34:2)	1.58±0.40	1.99±0.43[*]	1.50±0.40[#]	1.67±0.45
Serotonin	2.93±0.35	2.49±0.36[**]	2.37±0.30[**]	1.97±0.33[**, ##, &&]
Indole	5.31±0.65	4.54±0.45[**]	4.33±0.54[**]	3.67±0.56[**, ##, &&]
Glycohyodeoxycholic acid	1.51±1.86	3.11±1.65[*]	3.78±1.71[**]	2.34±0.80[&]
Deoxycholic acid	2.85±1.42	3.48±1.52	5.71±2.51[**, ##]	3.58±1.49[&&]
Tauro-α-Muricholic acid	3.83±1.35	1.38±0.56[**]	1.89±0.76[**]	1.32±0.36[**]
Taurocholic acid	9.47±4.61	2.71±0.73[**]	3.60±1.30[**]	2.64±1.05[**]
Taurohyodeoxycholic acid	4.48±1.53	1.80±0.62[**]	1.89±0.65[**]	1.53±0.57[**]
α-Muricholic acid	3.30±3.92	4.46±2.85	6.74±4.02[*]	3.56±1.80[&]
Cholic acid	4.76±4.07	6.48±2.93	9.07±4.02[**]	5.70±2.12[&]
Indoxyl sulfate	1.66±0.30	2.34±0.73[**]	2.05±0.62	1.36±0.31[##, &&]
1-Aminocyclopropanecarboxylic acid	5.12±0.64	6.09±0.93[**]	6.29±0.95[**]	5.81±0.92[*]
2,3-Dihydroxybenzoate	3.67±1.21	3.77±1.29	2.85±1.21	1.58±0.85[**, ##, &]
2-Hydroxy-2-Methylbutyric Acid	3.27±0.62	4.10±0.73[**]	3.48±0.77	3.31±0.77[#]
2-Hydroxybutyric acid	3.45±0.29	2.91±0.36[**]	3.15±0.55	2.89±0.34[**]
3-Amino-2-oxazolidone	2.04±0.22	1.86±0.12[*]	1.72±0.17[**]	1.50±0.26[**, ##, &]
3-Aminobutyric acid	7.46±0.89	7.17±1.05	6.44±0.84[**]	5.17±0.83[**, ##, &&]
4-Hydroxybenzenesulfonic acid	5.90±1.96	8.40±3.57[*]	8.21±2.76[*]	3.91±0.96[*, ##, &&]
4-Pyridoxic acid	8.59±1.96	9.33±2.66	7.60±2.87	6.44±2.46[*, ##]
5-Hydroxyindole	2.17±0.67	3.43±1.40[**]	2.66±0.96	1.53±0.47[##, &&]
DL-P-Hydroxyphenyl lactic acid	1.35±0.22	1.75±0.34[**]	1.80±0.39[**]	1.73±0.34[**]

Pantothenic acid	1.91±0.29	2.20±0.55	1.82±0.54$^{\#}$	1.55±0.30$^{*, \#\#}$
Piperidine	3.65±0.40	3.42±0.35	3.26±0.38*	2.84±0.29$^{**, \#\#, \&\&}$
Phosphoric acid	3.23±0.43	3.20±0.23	2.73±0.40$^{**, \#\#}$	2.88±0.31$^{*, \#}$
Benzophenone	16.88±6.02	10.53±3.50**	9.43±3.71**	7.56±3.69**
Glycolic acid	5.13±0.53	5.45±0.91	4.83±0.50$^{\#}$	4.51±0.64$^{*, \#\#}$
3-Hydroxybutyric acid	3.55±1.51	5.17±2.59	7.00±6.13	9.50±5.02$^{**, \#}$
Glycerol	7.80±2.03	9.96±2.84*	7.77±1.37$^{\#}$	11.14±1.98$^{**, \&\&}$
Uracil	1.31±0.39	1.75±0.59*	1.30±0.41$^{\#}$	1.15±0.32$^{\#\#}$
Threonic acid	5.86±1.74	7.52±2.64	10.17±5.02*	9.33±4.57*
Hypotaurine	5.94±1.09	7.16±2.84	5.14±1.14$^{\#}$	5.22±1.57$^{\#}$
Myo-Inositol	5.58±1.09	7.01±1.79**	5.26±0.53$^{\#\#}$	5.50±0.97$^{\#\#}$
Mannitol	5.15±2.80	5.39±2.75	2.11±1.12$^{**, \#\#}$	1.43±0.77$^{**, \#\#}$
Stearic acid	2.33±0.32	2.39±0.29	2.06±0.31$^{*, \#}$	2.50±0.33$^{\&\&}$
Cholesterol	6.78±1.82	10.33±2.75**	7.56±2.71$^{\#}$	9.76±1.90$^{**, \&}$
5-Hydroxytryptophan	4.00±4.93	6.07±4.88	2.56±2.69	8.13±6.42$^{\&}$
M-Cresol	7.77±4.76	17.75±8.61**	9.45±4.26$^{\#\#}$	7.43±2.71$^{\#\#}$
Heptanoic acid	8.50±0.86	9.17±1.18	9.73±1.67	10.24±1.71**
Trehalose	1.10±0.74	1.55±0.37	1.16±0.57	2.37±0.77$^{**, \#\#, \&\&}$
Galacturonic acid	5.50±0.98	7.51±2.39*	8.48±1.91**	8.43±1.87**
Glucuronic acid	1.59±0.29	2.65±0.37**	2.64±0.44**	2.87±0.51**
3-Aminoisobutyric acid	10.37±1.08	11.88±2.01*	10.40±1.13$^{\#}$	10.56±0.71$^{\#}$
Ribose	5.17±2.53	9.53±6.23**	4.34±1.92$^{\#\#}$	6.77±2.19
Xylitol	6.24±0.70	6.72±1.58	5.40±0.89$^{\#\#}$	6.03±0.77
Ribose-5-phosphate	7.03±1.34	8.74±2.03**	6.65±0.59$^{\#\#}$	6.92±1.19$^{\#\#}$
Glutamine	2.90±0.55	3.48±0.83	3.20±0.80	4.04±0.82$^{**, \&}$

3-Indoleacetic acid	2.79±0.98	2.64±1.12	2.32±0.50	1.71±0.70$^{**, \#}$
3-Indolepropionic acid	2.15±0.60	2.53±0.55	1.48±0.41$^{*, \#\#}$	1.80±0.92$^{\#}$
β-Linoleic acid	6.57±0.77	6.95±1.20	7.07±1.05	8.05±1.26$^{**, \#, \&}$
FFA22:6	6.20±1.38	7.49±0.79	7.53±1.84	8.82±2.44**
Urea	6.05±0.48	5.63±1.08	4.64±1.29*	3.49±0.60$^{**, \#\#}$

Note: *n* represent the number of samples. LSD analysis. Compared with Control, $^{*}p<0.05$, $^{**}p<0.01$; Compared with 10Gy, $^{\#}p<0.05$, $^{\#\#}p<0.01$; Compared with 20Gy, $^{\&}p<0.05$, $^{\&\&}p<0.01$.

Table S8: Correlation analysis of differential metabolites in plasma and lung tissues at 5d after WTI.

Number	Compound Name	Correlation Coefficient（r）	p Value
1	Acylcarnitine C9:1	0.766	0.000
2	Tauro-α-Muricholic acid	0.743	0.000
3	Taurocholic acid	0.740	0.000
4	Acylcarnitine C5:0	0.719	0.000
5	Urea	0.676	0.000
6	Acylcarnitine C20:0	0.568	0.000
7	Taurohyodeoxycholic acid	0.540	0.001
8	Acylcarnitine C20:1	0.505	0.002
9	Leucine	0.378	0.023
10	Asparagine	0.332	0.048

Note: Pearson or Spearman correlation analysis.

Table S9: Stimulation of RILI classification after WTI.

Given dose（Gy）	0	10	20	35
Exposure dose（Gy）	<6	6-16	16-26	>26
Triage	Control	Mild	Moderate	Severe

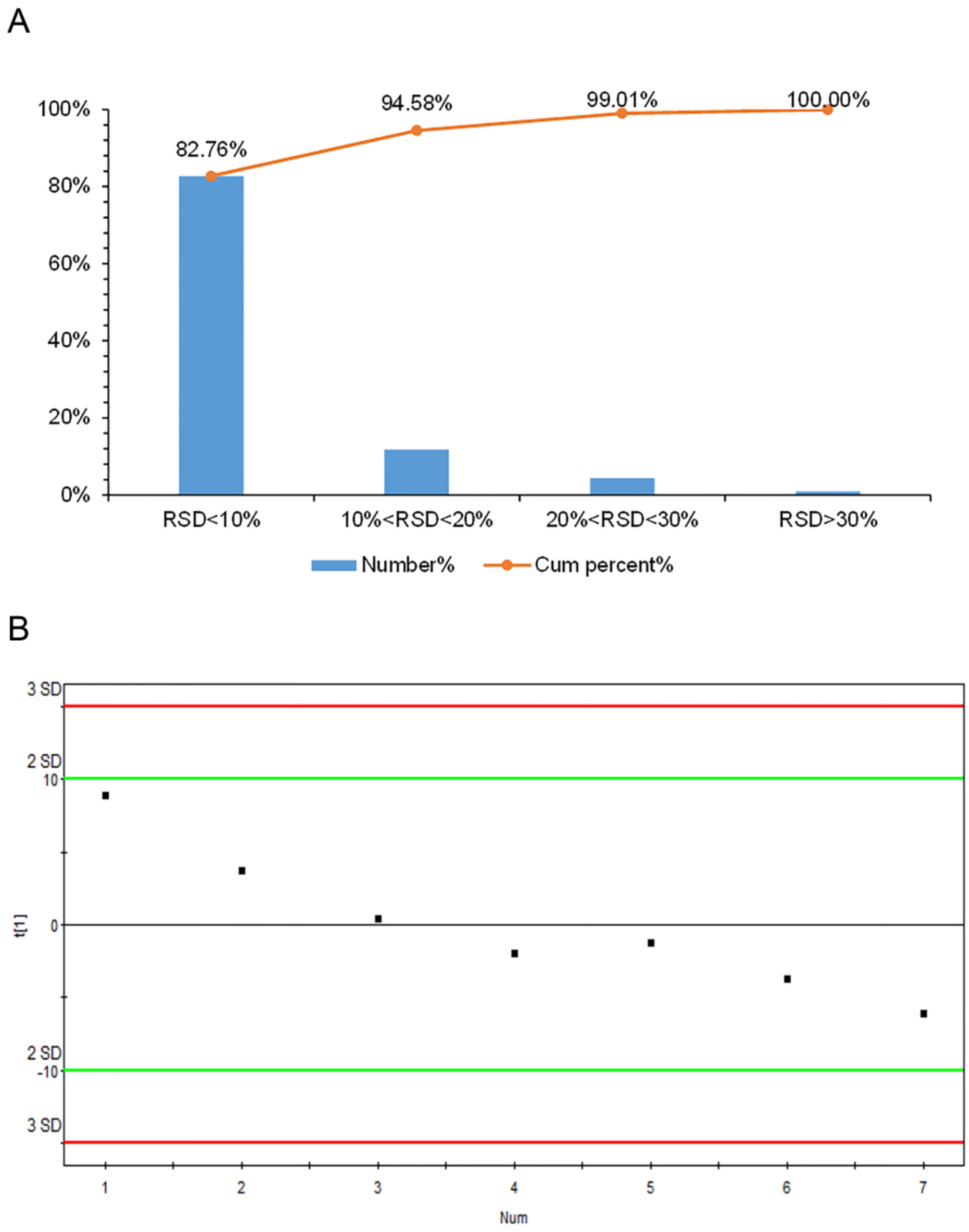

Figure S1. Reproducibility and stability of metabolic profiling in lung tissues were assessed. (**A**) RSD distribution of the QC samples. (**B**) PCA score plots of the QC samples distributions in LC-MS.

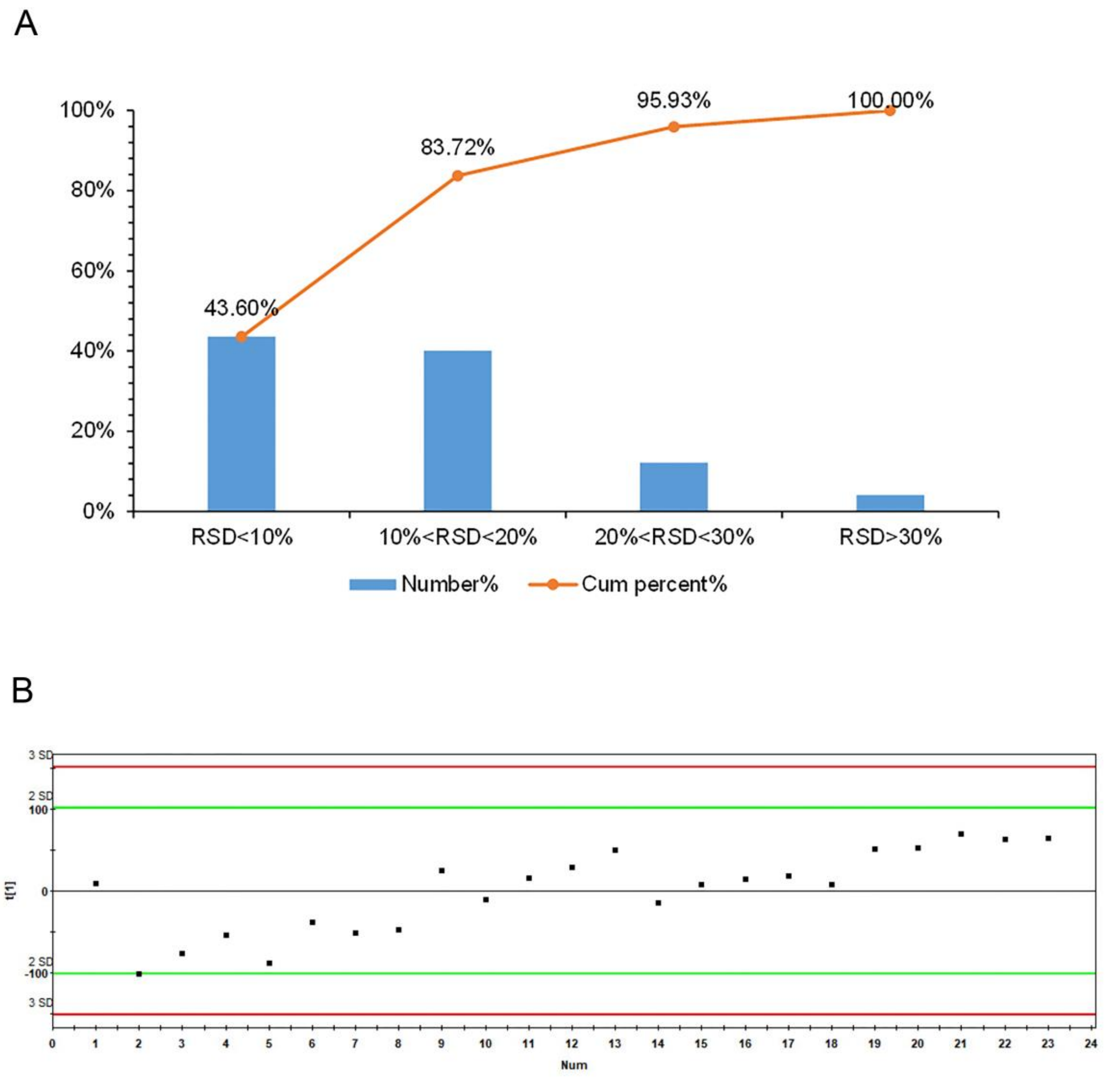

Figure S2. Reproducibility and stability of metabolic profiling in plasma were assessed. (**A**) RSD distribution of the QC samples. (**B**) PCA score plots of the QC samples distributions in LC-MS.

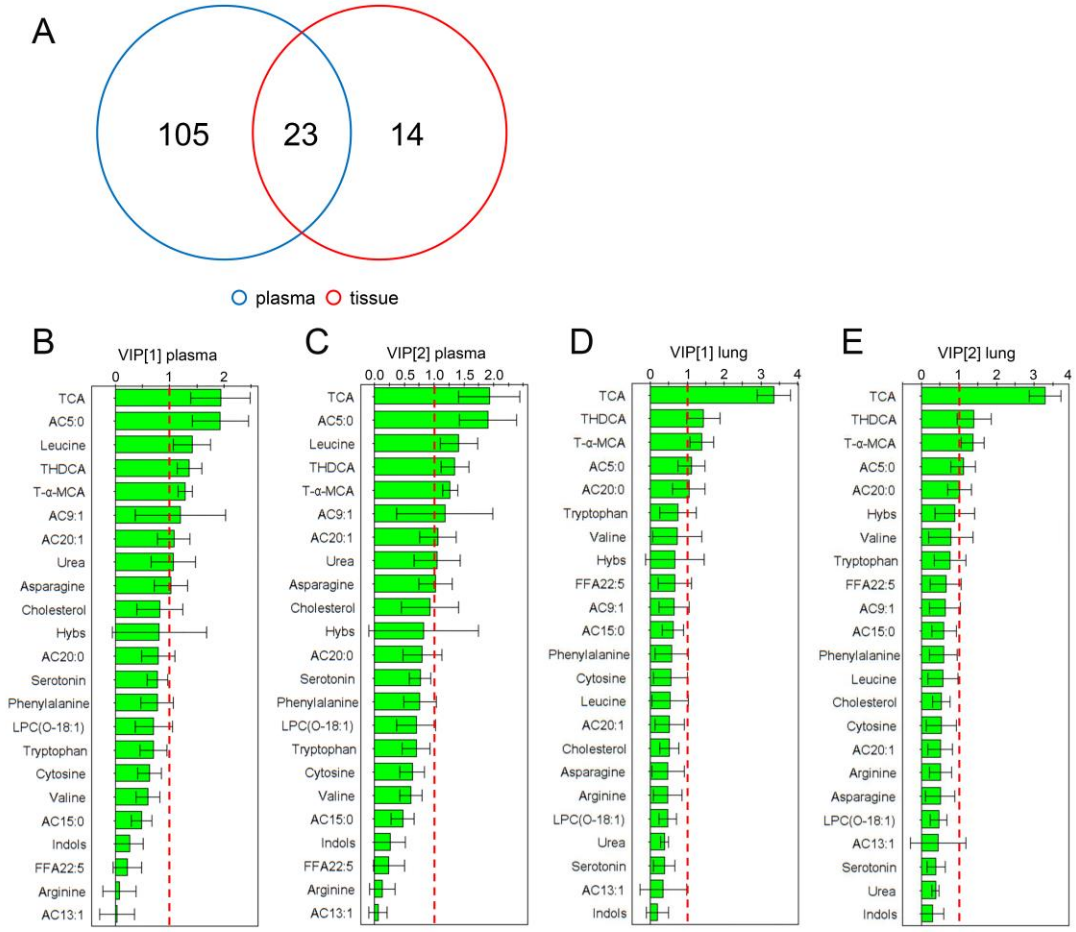

Figure S3. Screen of biomarkers in plasma. (**A**) Venn diagram showing the shared and unique metabolites between plasma and lung tissues. (**B, C, D, E**) Column plots of VIP value in plasma and lung tissues, metabolites with VIP [1] and VIP [2] > 1 were selected.

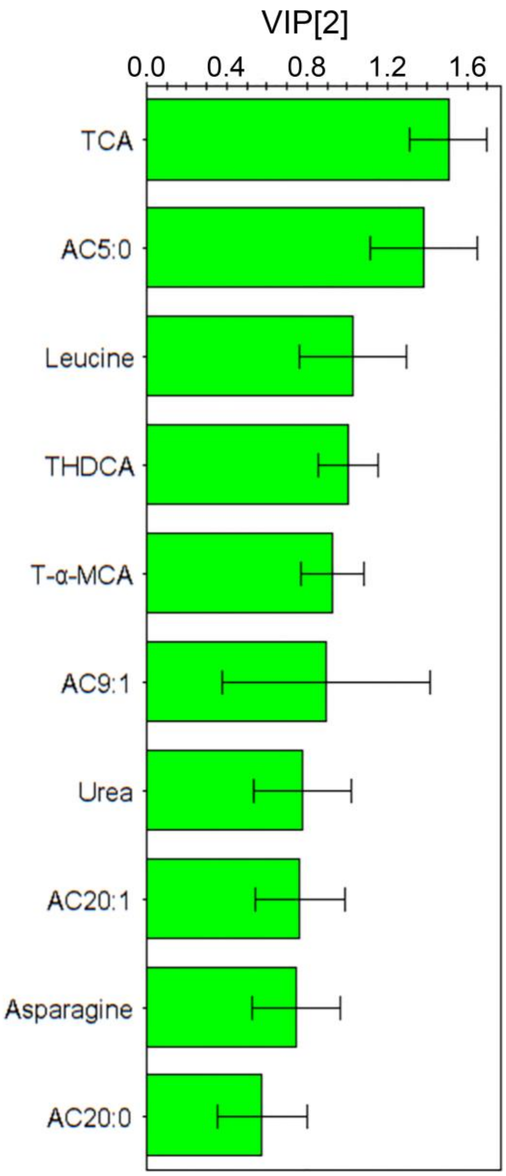

Figure S4. Column plots of VIP value in plasma, metabolites with more importance were selected as biomarkers.

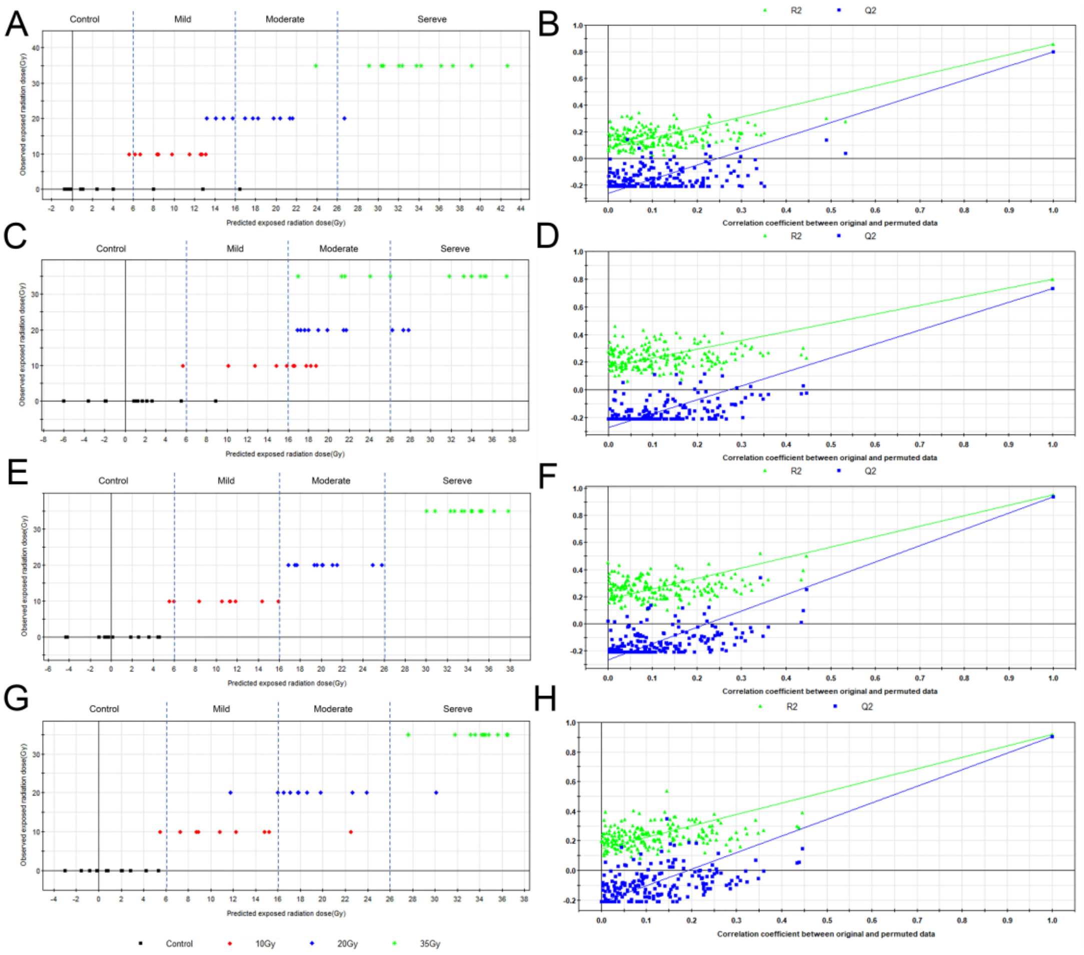

Figure S5. (**A, C, E, G**) Comparison between the predicted radiation doses and observed values in plasma at 1d, 2d, 3d and 5d after WTI. (**B, D, F, H**) Validation plots obtained from 200 permutation tests based on the panel of these 7 biomarkers, and the model parameters showed the predictive ability of model.

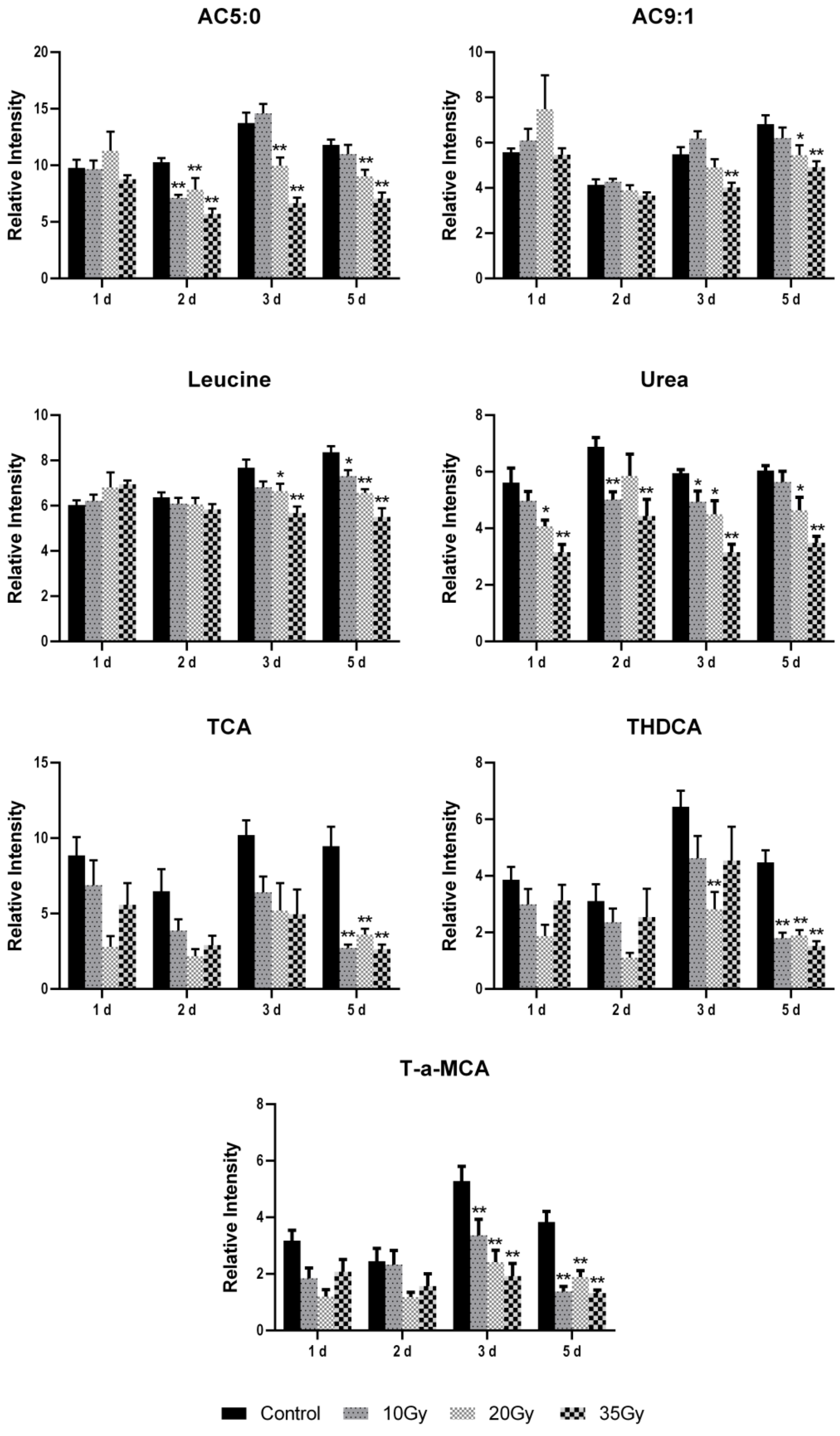

Figure S6. Dynamic changes of these 7 potential biomarkers in plasma. Compared with Control, *$p<0.05$, **$p<0.01$.

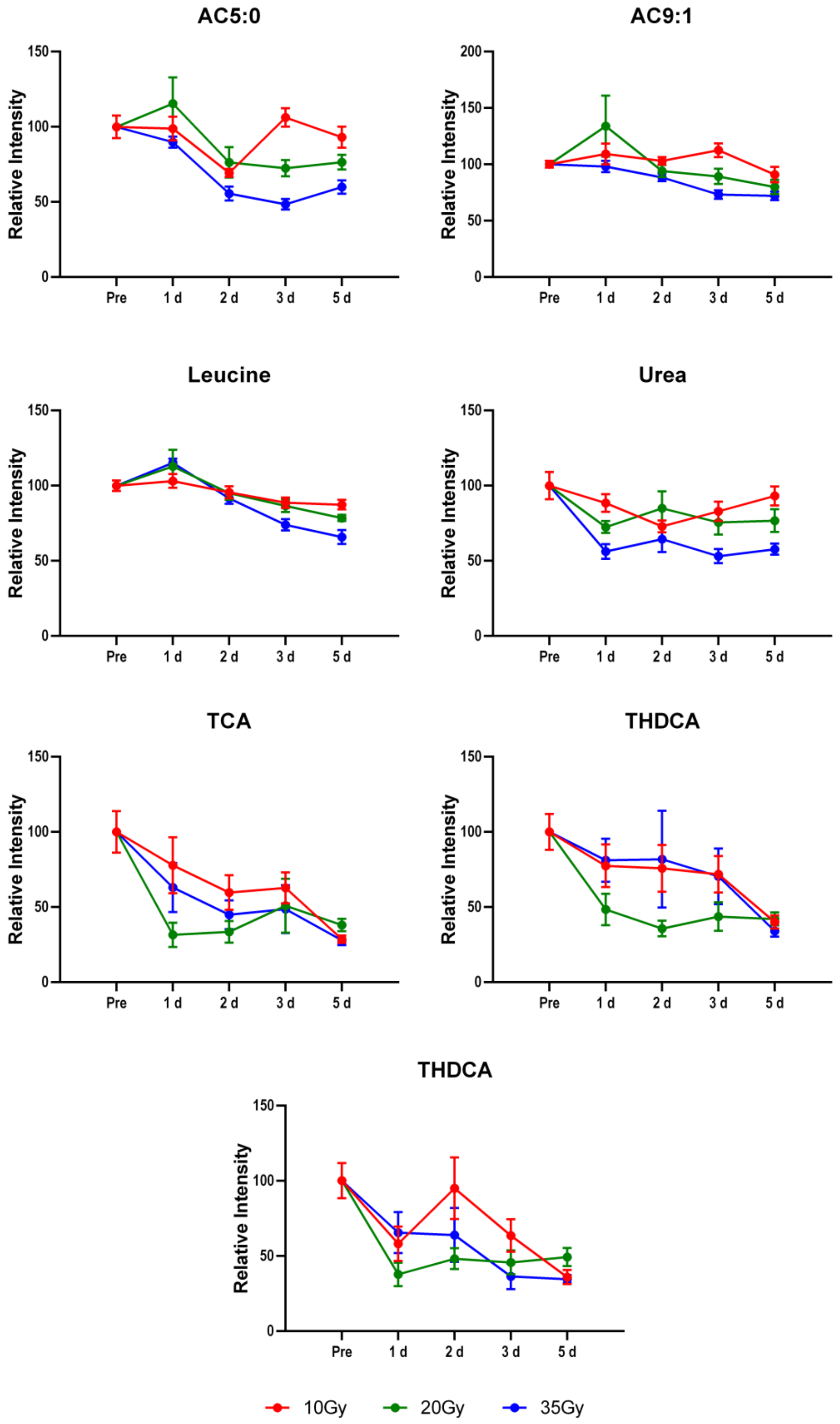

Figure S7. Dynamic changes of these 7 potential biomarkers in plasma (normalized by control group).

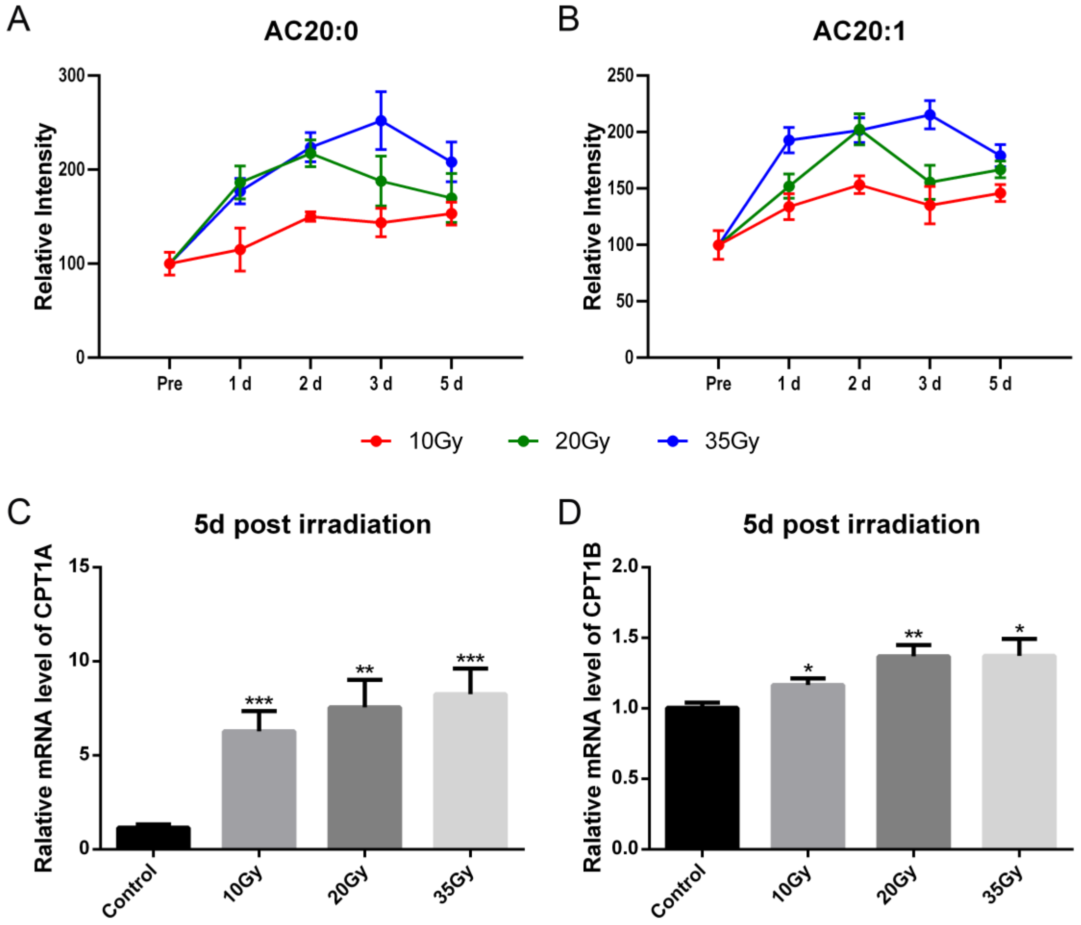

Figure S8. (**A, B**) Dynamic changes of Acylcarnitine C20:0 and Acylcarnitine C20:1 in plasma (normalized by control group). (**C, D**) The mRNA level of CPT1A and CPT1B in lung tissues at 5d after different radiation doses exposure. Compared with Control, $^{*}p<0.05$, $^{**}p<0.01$, $^{***}p<0.001$.

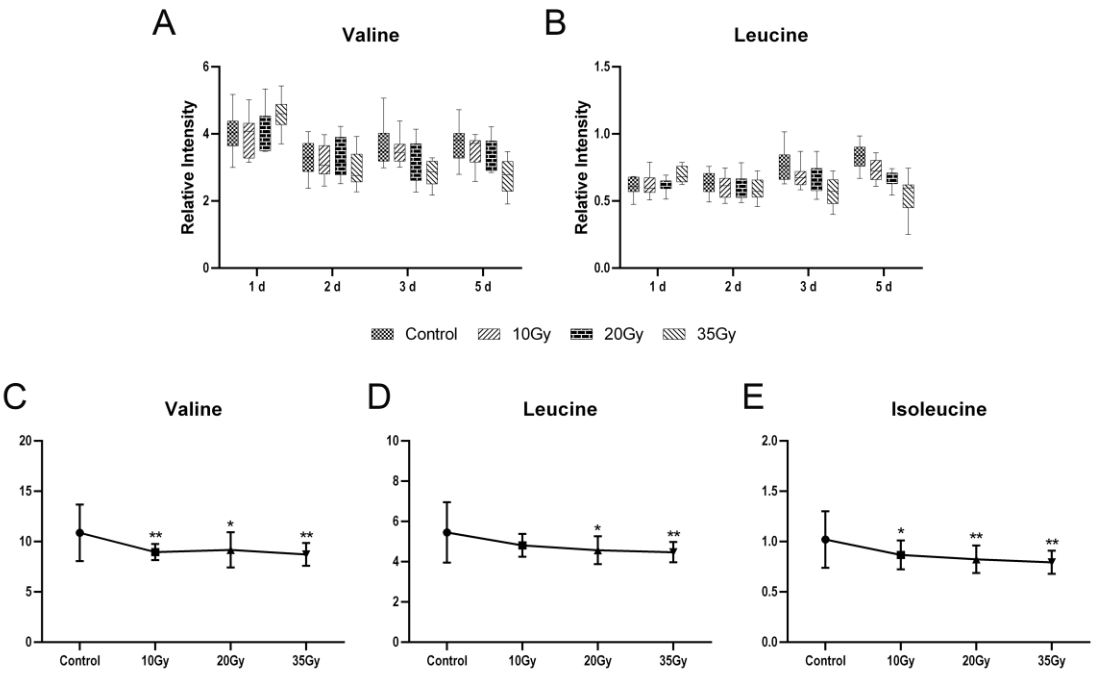

Figure S9. (**A, B**) Dynamic changes of valine and leucine in plasma. (**C, D, E**) Changes of valine, leucine and isoleucine in lung tissues. Compared with Control, *p<0.05, **p<0.01.

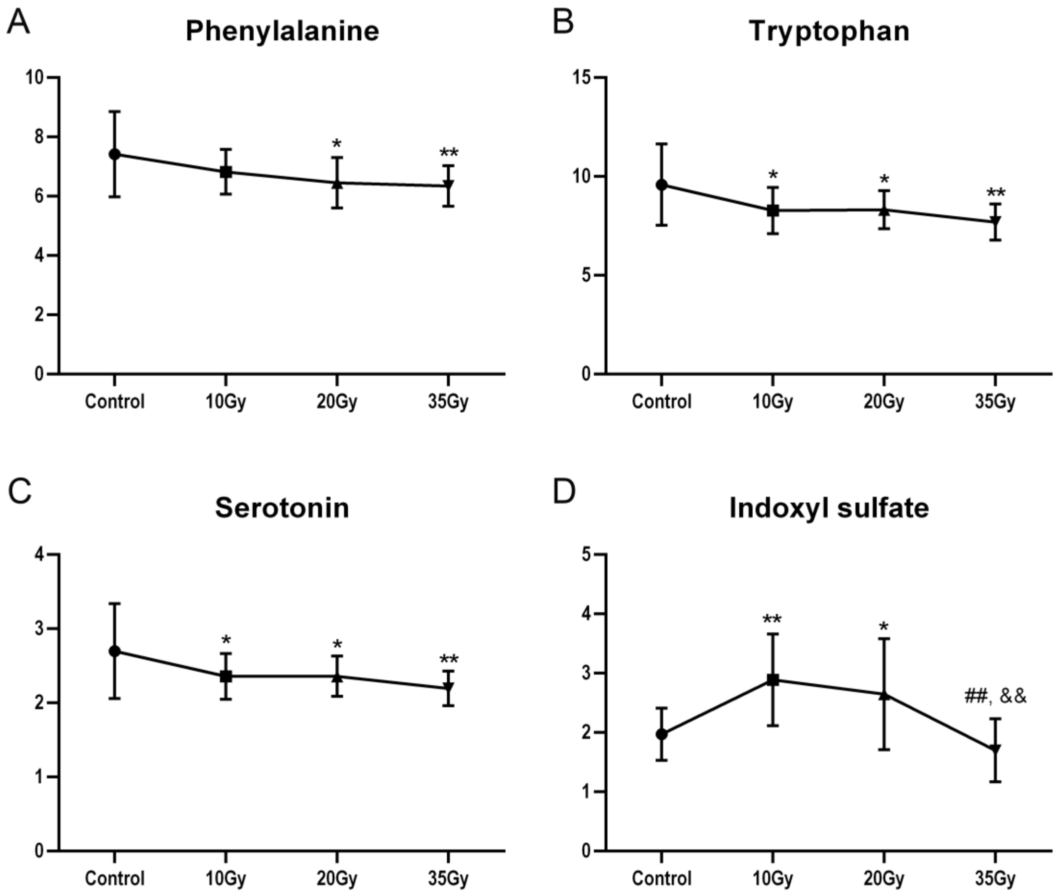

Figure S10. (**A, B**) Changes of aromatic amino acids (phenylalanine and tryptophan) in lung tissues. (**C, D**) Changes of Tryptophan-related metabolites (serotonin and indoxyl sulfate) in lung tissues. Compared with Control, *p<0.05, **p<0.01; Compared with 10Gy, $^{\#\#}$p<0.01; Compared with 20Gy, $^{\&\&}$p<0.01.

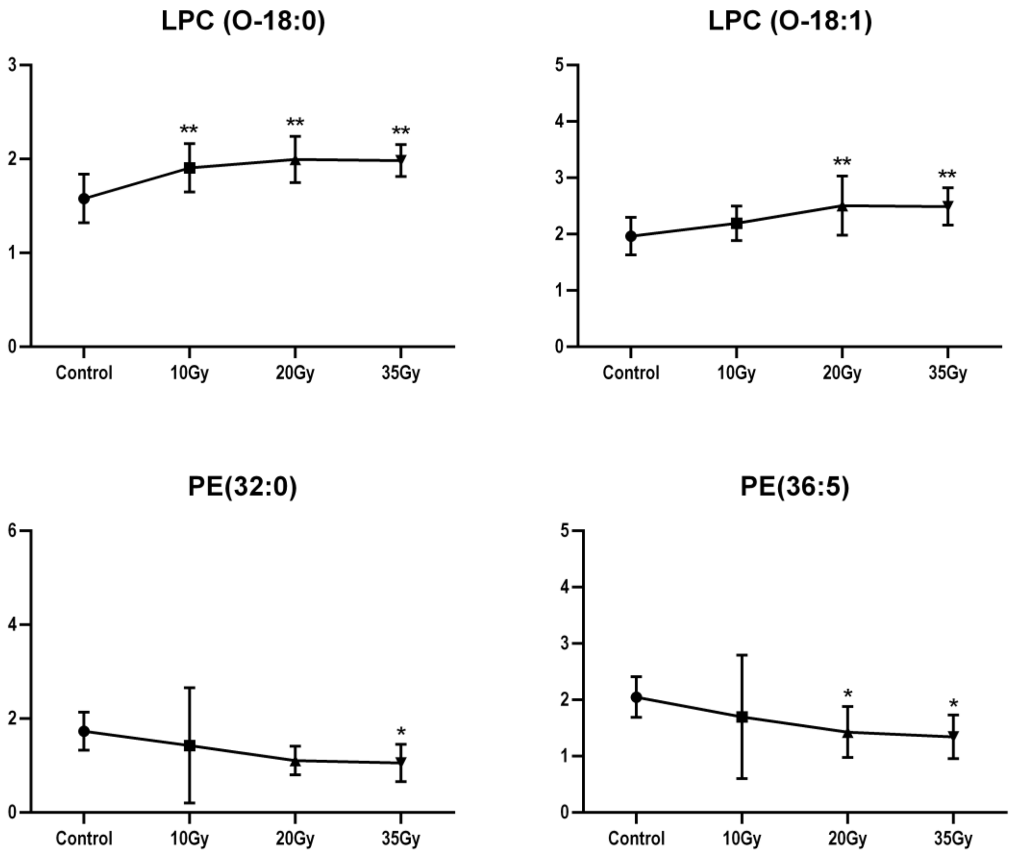

Figure S11. Changes of lipids (LPC (0-18:0, LPC (O-18:1), PE (32:0) and PE (36:5)) in lung tissues. Compared with Control, *p<0.05, **p<0.01.
